# Synthesis and pharmacological characterization of ethylenediamine synthetic opioids in human μ‐opiate receptor 1 (OPRM1) expressing cells

**DOI:** 10.1002/prp2.511

**Published:** 2019-08-22

**Authors:** Tom Hsu, Jayapal R. Mallareddy, Kayla Yoshida, Vincent Bustamante, Tim Lee, John L. Krstenansky, Alexander C. Zambon

**Affiliations:** ^1^ Department of Biopharmaceutical Sciences, School of Pharmacy and Health Sciences Keck Graduate Institute Claremont California

**Keywords:** biased signaling, Gα_i_ signaling, internalization, synthetic opioids, μ‐opioid receptor (OPRM1)

## Abstract

Opioids are powerful analgesics acting via the human μ‐opiate receptor (hMOR). Opioid use is associated with adverse effects such as tolerance, addiction, respiratory depression, and constipation. Two synthetic opioids, AH‐7921 and U‐47700 that were developed in the 1970s but never marketed, have recently appeared on the illegal drug market and in forensic toxicology reports. These agents were initially characterized for their analgesic activity in rodents; however, their pharmacology at hMOR has not been delineated. Thus, we synthesized over 50 chemical analogs based on core AH‐7921 and U‐47700 structures to assess for their ability to couple to Gα_i_ signaling and induce hMOR internalization. For both the AH‐7921 and U‐47700 analogs, the 3,4‐dichlorobenzoyl substituents were the most potent with comparable EC_50_ values for inhibition of cAMP accumulation; 26.49 ± 11.2 nmol L^−1^ and 8.8 ± 4.9 nmol L^−1^, respectively. Despite similar potencies for Gα_i_ coupling, these two compounds had strikingly different hMOR internalization efficacies: U‐47700 (10 μmol L^−1^) induced ~25% hMOR internalization similar to DAMGO while AH‐7921 (10 μmol L^−1^) induced ~5% hMOR internalization similar to morphine. In addition, the *R*, *R* enantiomer of U‐47700 is significantly more potent than the *S*, *S* enantiomer at hMOR. In conclusion, these data suggest that U‐47700 and AH‐7921 analogs have high analgesic potential in humans, but with divergent receptor internalization profiles, suggesting that they may exhibit differences in clinical utility or abuse potential.

**Abbreviations**
GPCRs: G protein‐coupled receptors
GRK: G protein-coupled receptor kinase
hMOR: human μ‐opiate receptor
HRMS: high resolution mass spectrometry
HPLC: high-performance liquid chromatography
MOR: μ‐opiate receptor
NLX: naloxone
NMR: nuclear magnetic resonance

## 1 INTRODUCTION

The ability of opiates to suppress painful stimuli is undisputed. Opiates are a class of small molecules or peptides regardless of structure that can bind and activate opiate receptors. Morphine is an opioid extracted from the opium poppy that has been used as early as the third century BC to treat dysentery, pain, and suffering.^1^ It relieves pain by binding to and activating any of the three gene products encoding for cell surface G protein‐coupled receptors (GPCRs). These receptors are the μ‐, δ‐, or κ‐opiate receptors and they are encoded by the OPRM1, OPRD1, and OPRK1 genes, respectively.^2^, ^3^ Opioid analgesic properties stem from opioid receptor gene expression in sensory neurons of the brain and peripheral CNS and their coupling to intracellular heterotrimeric G proteins. Opiate binding induces a conformational change in opiate receptors and signals to rapidly suppress neuronal excitability by G protein‐dependent modulation of Ca^2+^, K^+^, and Na^+^ currents resulting in a profound reduced perception of pain. Of the three main opiate receptor subtypes, only compounds with relatively high selectivity for the μ‐opiate receptor (MOR) have achieved widespread clinical utility due, in part, to increased adverse effects such as dysphoria, convulsions, or poor selectivity of agents that have been developed to selectively target the δ‐ or κ‐ opiate receptors.^1^, ^2^

Agonist binding‐induced conformational changes of the MOR, in addition to activating inhibitory Gα_i_ proteins, cause the phosphorylation of intracellular residues such as Ser^375^ by a number of kinases (eg G protein‐coupled receptor kinases [GRKs], PKC).^4^ The MOR phosphorylation sites and the efficacy of phosphorylation can differ based on the agonist structure. MOR phosphorylation leads to β‐arrestin recruitment, receptor desensitization, and internalization which are all regulatory processes central to the development of opiate tolerance.^5^, ^6^ While many synthetic and naturally occurring MOR agonists have high potency and efficacy for coupling to Gα_i_, they can diverge significantly in their ability to promote β‐arrestin recruitment and receptor internalization.^7^, ^8^, ^9^ For example, despite morphine's high potency and efficacy for Gα_i_ coupling and widespread clinical use, it has very low efficacy for β‐arrestin recruitment and causes very little MOR internalization.^7^ Conversely, endogenously produced opioids, such as the enkephalins and β‐endorphins, are within a 10‐fold range of morphine in terms of Gα_i_ coupling potency; however, they are far more efficacious than morphine for recruiting β‐arrestin and inducing receptor internalization.^9^

The concept that agonists could be designed to preferentially couple to Gα_i_ vs β‐arrestin recruitment, referred to as “biased signaling,” was a driving force behind the development of new opiates such as oliceridine.^10^ This idea was bolstered by reports showing that β‐arrestin‐2 knockout mice display increased analgesia, decreased tolerance, and have less respiratory depression after morphine administration.^11^, ^12^, ^13^ Hence new opiates, such as oliceridine, were designed and selected for the ability to couple strongly to the Gα_i_ pathway but with low efficacies for β‐arrestin recruitment and internalization, similar to morphine. However, recent events, such as the failure of FDA approval for oliceridine in 2018 and new studies using mouse models with targeted mutations in the OPRM1 gene that prevent MOR internalization, suggest that this may not be the best approach for developing safer opiates with fewer side effects. Mutations of carboxyl tail serine and threonine residues, including Ser^375^, that are phosphorylated by GRKs reveal that respiratory depression and constipation are significantly exacerbated when MOR receptors fail to internalize after agonist binding^14^ while analgesic tolerance is reduced. Thus, perhaps the pursuit of new opioids that better mimic the endogenous system might result in novel more “balanced” agents with improved clinical utility over morphine‐like derivatives.

In light of this, we sought to synthesize and pharmacologically characterize a series of structural analogs based on the ethylenediamine structural analogs AH‐7921 and U‐47700 and compare their pharmacology to morphine and the endogenous opioid mimetic DAMGO. These two synthetic opioids, first synthesized and patented in the early 1970s, have naloxone (NLX)‐reversible analgesic potential in rodent models^15^, ^16^ in the potency range of morphine. However, their pharmacological properties, including their ability to cause internalization of *human* μ‐opioid receptors (hMORs), are unknown. Thus, they represent a potentially useful series of core structures that are relatively easy to synthesize from where new “balanced” or “biased” opiates could be designed.

The design of analogs was to assess both compounds described in the patents, as well as related novel analogs, for their pharmacological selectivity and efficacy for causing hMOR internalization. Additionally, while the prior literature on the U‐series compounds indicated that stereoisomers differ in their selectivity for κ‐ vs μ‐opioid receptors, we sought to more clearly define the impacts of these differences on hMOR pharmacology by synthesizing and testing single stereoisomers of the U‐series compounds. We present herein findings related to structure activity relationships of these two compounds and over 50 structural analogous to provide new insights on how chemical structures affect potency for Gα_i_ signaling and efficacy for hMOR internalization.

## 2 MATERIALS AND METHODS

### 2.1 Synthesis of compounds

The achiral amine precursor 1‐aminomethyl‐1‐cyclohexanedimethylamine for the AH‐series compounds, **A01‐17,** was prepared from cyclohexanone according to the original patent US4049663 Example 1b.^17^ (1*R*,2*R*)‐N,N,N′‐trimethyl‐1,2‐diaminocyclohexane, the precursor for the *R,R* enantiomer of the U‐series compounds, **U01‐17**, (1*R*,2*R*)‐N,N‐dimethyl‐1,2‐diaminocyclohexane, the precursor for the amide desmethyl U‐series compounds, **Udes01‐09**, and (1*S*,2*S*)‐N,N,N'‐trimethyl‐1,2‐diaminocyclohexane, the precursor for the *S,S* enantiomer of the U‐series compounds, **US01‐09**, were obtained from LabNetwork (San Diego, CA). The acid chlorides used were purchased from Fisher Scientific (analog code numbers **01‐04**, **07‐17**) or Sigma Aldrich (analog code numbers **05** and **06**).

### 2.2 Acylation of starting amines

The respective acid chloride (1.05 eq) was added to a solution of the appropriate precursor amine (1.0 eq) and triethylamine (1.0 eq) in 5 mL of dry diethyl ether and stirred at room temperature for 16 hour. The reaction mixture was extracted with ethyl acetate (3×), washed with brine, dried over Na_2_SO_4_, and concentrated in vacuo. The crude product was recrystallized from dichloromethane or precipitated with ethyl acetate upon sonication to give desired product as a solid. Reported hydrochloride salts of final compounds were made using 2.0 N HCl in diethyl ether solution.

### 2.3 Validation of synthesized compound purity and structure

Nuclear magnetic resonance (NMR) spectra were recorded on a Varian 400MR spectrometer (proton frequency 399.765 MHz) equipped with an AutoX DB broadband probe. Pulse sequences, acquisition, and data processing were accomplished using VnmrJ software (VnmrJ 4.2, Agilent Technologies, Santa Clara, CA). The spectrometer was locked on to D_2_O and spectra were acquired at 28°C without spinning. Water suppression suitability studies were carried out using the presaturation (presat), WET (WET1D), and excitation sculpting (water_ES) pulse sequences (VnmrJ 4.2, Agilent Technologies, Santa Clara, CA) with automatic suppression of the tallest peak (water at δ 4.86 ppm), an observation pulse of 90° (10.8 μs), a spectral width of 6410.3 Hz, a relaxation time of 30 second, and an acquisition time of 5.112 second. Eight scans were taken. Three replicates were taken for each sample.

High Resolution Mass Spectrometry spectra were collected on a Agilent Technologies 6530 Accurate‐Mass Q‐TOF LC/MS spectrometer using direct infusion into the nanoelectronspray source. Samples were dissolved in high‐performance liquid chromatography (HPLC)‐grade methanol to a final concentration of ∼0.01 mg mL^−1^. Spectra were run with 0.1% (v/v) formic acid/ HPLC‐grade methanol as solvent.

Purity determinations were performed by GCMS using a Shimadzu GC/MS 2010 SE with an Rtx‐5MS column (a DB‐5 MS equivalent); 30 m × 0.25 mm × 0.25 m. The carrier gas was helium at 1 mL min^−1^, with the injector at 280°C, MSD transfer line at 280°C, and ion source at 200°C. Injection Parameters: Split Ratio = 1:15, 1 μL injected. MS Parameters: Mass scan range: 34‐550 amu & Threshold: 100. Acquisition mode: scan. The oven programs were as follows: (a) 90°C initial temperature for 2.0 minutes; (b) Ramp to 300°C at 14°C min^−1^; (c) Hold final temperature for 10.0 minutes.

### 2.4 DNA constructs

Human µ‐opioid receptor, OPRM1, with three sequential hemagglutinin antigen (3xHA) tags at the N‐terminus (cDNA Resource Center OPRM10TN00) was subcloned into pENTR/D‐TOPO vector (Thermo Fisher K240020). The subcloned plasmid underwent a three‐way Gateway LR recombination along with pENTR‐EF1α vector containing EF1α promoter and the lentiviral 2k7bsd destination vector with blasticidin resistance coding sequence, as described previously.^18^ The resulting lentiviral plasmid vector is referred to herein as EF1α‐3xHA‐OPRM1‐2k7bsd.

### 2.5 Cell lines

Human fibrosarcoma HT1080 cells (ATCC CCL‐121) are maintained in DMEM high glucose, supplemented with 10% fetal bovine serum, 1% penicillin/streptomycin, 1% L‐glutamine, 1% nonessential amino acid, and 1% sodium pyruvate. Wild type (WT) HT1080 cells were transduced with lentivirus generated with EF1α‐3xHA‐OPRM1‐2k7bsd lentiviral plasmid and transduced cells were selected for using DMEM supplemented media with 10 µg mL^−1^ blasticidin.

### 2.6 Western blot

5 × 10^5^ OPRM1‐expressing HT1080 cells were lysed with 1xLDS buffer directly and ~25 μg of protein lysate is loaded into NuPAGE 4%‐12% Bis‐Tris Protein Gels (Thermo Fisher NP0335BOX) and transferred onto PVDF membrane. Membrane was blocked in 1X TBST with 5% w/v nonfat dry milk for 1 hour and probed with either rabbit anti‐HA antibody (Cell Signaling 3724S) or rabbit anti‐GAPDH (Cell Signaling 2118S) overnight at 4°C. The membrane was washed 3x with 1X TBST and probed with goat anti‐rabbit HRP antibody (Santa Cruz SC2004), then visualized using SuperSignal West Femto Maximum Sensitivity Substrate (Thermo Fisher 34095) in Azure Biosystems c500 imaging system.

### 2.7 Immunocytochemistry

OPRM1‐expressing or WT HT1080 cells were seeded at 7.5 × 10^5^ onto cover glass precoated with 0.1 mg mL^−1^ Poly‐D‐Lysine (Sigma P7280). Cells were first washed with Hank's Balanced Salt Solution (Fisher Scientific MT21022CV), and then fixed with 4% paraformaldehyde/PBS for 15 minutes followed by quenching with 0.1 mol L^−1^ glycine for 15 minutes. Afterward, the cells are washed 3x with 1X PBS and then blocked in 1%BSA/TBST for 1 hour. Rabbit monoclonal anti‐HA antibody (Cell Signaling 3724S) is used to stain the cells overnight, followed by goat anti‐rabbit Alexa Fluor 488 (Thermo Fisher A11070) and counterstained with Draq5 (Biolegend 424101) at 1:200 dilution. Slides were mounted and visualized using Leica DMI6000 confocal microscope using 63× oil‐immersion objective, with image processing using ImageJ software.

### 2.8 Catchpoint cAMP assay

OPRM1‐expressing or WT HT1080 cells were seeded at 2 × 10^4^ in 96‐well plates in DMEM high glucose, supplemented with 5% fetal bovine serum, 1% penicillin/streptomycin, 1% L‐glutamine, 1% nonessential amino acid, and 1% sodium pyruvate. Culture media was removed and cells were washed with Krebs‐Ringer Bicarbonate Buffer (Sigma K4002), pH 7.4, then 1 mmol L^−1^ IBMX (Sigma I5879) was added to cells, and incubated at 37°C for 30 minutes. Compounds were added and incubated at 37°C for 15 minutes, followed by addition of 100 µmol L^−1^ forskolin (FSK) (Sigma F3917). Selected samples were treated with 10 μmol L^−1^ NLX (Sigma N7758) in addition to analogs that significantly altered FSK‐induced cAMP levels to validate that any observed changes in cAMP levels were mediated directly by hMOR. Cells were further incubated at 37°C for 15 minutes. A solution of 1X PBS was used for untreated cells in place of opioid compounds, FSK, and NLX as a vehicle control. All solutions were removed and the cells were lysed with Catchpoint Cyclic‐AMP Fluorescent Assay Kit Lysis Buffer (Molecular Devices R8088) and shaken on a plate shaker for 15 minutes at room temperature. Lysed cells are processed according to manufacturer's protocol in the Catchpoint Cyclic‐AMP Fluorescent Assay Kit and the cAMP levels were measured by SpectraMax Gemini EM Microplate Reader.

### 2.9 Quantification of agonist‐mediated receptor internalization

OPRM1‐expressing cells were seeded at 7.5 × 10^5^ in 6‐well plates. Culture media was removed and drug compounds diluted in 10% FBS DMEM high glucose media (fully supplemented) were added to the cells. The drug compound media was removed following 60 minutes incubation at 37°C. Cells were washed with ice‐cold PBS (Ca^2+^ and Mg^2+^ free) and kept cold (4°C) for the remainder of the assay to prevent further receptor trafficking. Washed cells were detached from the plate by incubating with 2.9 mmol L^−1^ EDTA in PBS for 30 minutes. Detached cells were pelleted and washed with cold PBS before incubation with rabbit anti‐HA antibody (Cell Signaling 3724S) at 4°C. Cells are centrifuged down and washed with cold PBS before incubation with goat anti‐rabbit Alexa Fluor 488 antibody (Invitrogen A11070) at 4°C. Cells are then centrifuged and washed with cold PBS before analysis on BD Accuri C6 Flow Cytometer. Flow cytometry data were analyzed using FlowJo V10 software and gated for single, viable cells.

### 2.10 Data analysis and statistics

All data are presented as mean ± SEM. Due to the high number of compounds tested, for the initial screening of compounds, each compound was tested at three different concentrations (0.01, 0.1, and 1 μmol L^−1^) in triplicate using the Catchpoint cAMP assay on two independent experiments (n = 2). In addition, a morphine sulfate (MS) group was included as an internal positive control in each independent experiment (data not shown) in addition to a 1 μmol L^−1^ compound plus 10 μmol L^−1^ NLX group to validate that any changes in cAMP levels were mediated by hMOR. Cyclic AMP responses for each of the three single doses were compared to FSK alone by one‐way ANOVA with Dunnett multiple comparison (GraphPad Prism 7). An adjusted *P* < 0.05 being considered significant. All high potency agonists, that is, those which showed significant suppression of cAMP at the 0.01 µmol L^−1^ compared to FSK‐only (Dunnett's adjusted *P* < 0.05) dose, were then tested with expanded dose ranges (in triplicate) to compute the average EC_50_ value ± SEM from three independent expanded dose range experiments (n = 3). The EC_50_ values for each experimental replicate were computed by fitting dose response data for each experimental replicate to a variable‐slope sigmoidal curve (GraphPad Prism 7). Receptor internalization data were analyzed in GraphPad Prism 7 using unpaired *t* tests.

## 3 RESULTS

### 3.1 Chemical syntheses, stereochemical considerations, and structural analog design

**Figure 1:**
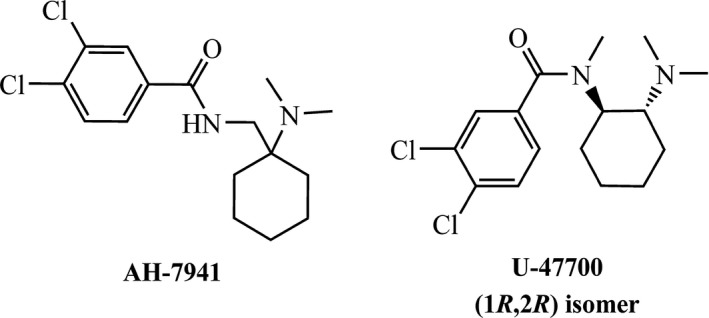
Structures of AH‐7921 and U‐47700

**Figure 2:**
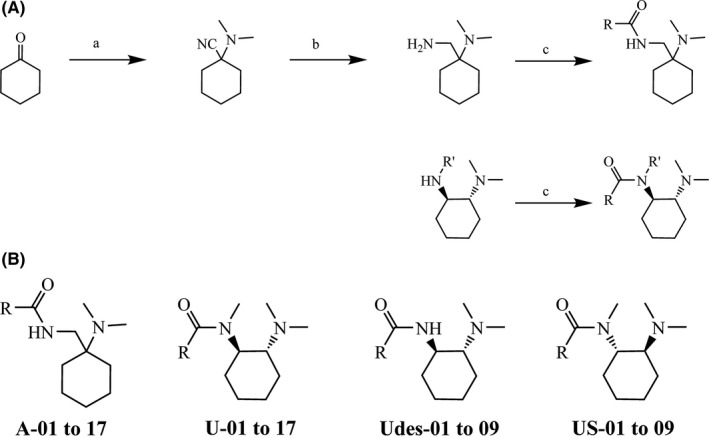
Core analog synthesis and structure overview (A) Synthetic routes to all analogs (R′ = Me for U‐ & US‐series, R′ = H for Udes‐series): (a) KCN, Me2NH.HCl, H2O, rt, 16 hour; (b) LiAlH4, Et2O, rt, 16 hour; (c) Et3N, RCOCl, Et2O, rt, 16 hour. US starts with the (1*S*,2*S*) diamine. (B) General structures of the analogs in the four series tested: A, U, Udes, and US

AH‐7921 (Figure 1) is an achiral compound made from cyclohexanone via a Strecker reaction as described in the original patents and in (Figure 2A).^17^, ^19^, ^20^ The key intermediate of aminomethyl‐1‐cyclohexanedimethylamine was used to synthesize AH‐7921 (Compound **A01**) and derivatives. U‐47700 (Figure 1) has two chiral centers but is commercially available only as a racemic *trans*‐1,2‐diaminocyclohexane mixture. The goal of this study was to study the stereoselective pharmacology of single (*R*,*R*) or (*S*,*S*) isomers of U‐47700 and related analogs, which had not previously been determined. Thus, for the synthesis of (*R*,*R*)‐isomer U‐series (**U01‐17,** Figure 2B) and the Udes‐series (**Udes01‐09**, Figure 2B) compounds, the (1*R*,2*R*)‐N,N,N'‐trimethyl‐1,2‐diaminocyclohexane and (1*R*,2*R*)‐N,N‐dimethyl‐1,2‐diaminocyclohexane were used, respectively, as the starting material. The (*S*,*S*)‐isomer US‐series (**US01‐09**) was synthesized using (1*S*,2*S*)‐N,N,N'‐trimethyl‐1,2‐diaminocyclohexane as the starting material. (Analytical data of all compounds available in Supplementary information).

### 3.2 Validation of 3xHA‐tagged hMOR expression in HT1080 fibrosarcoma cells

**Figure 3:**
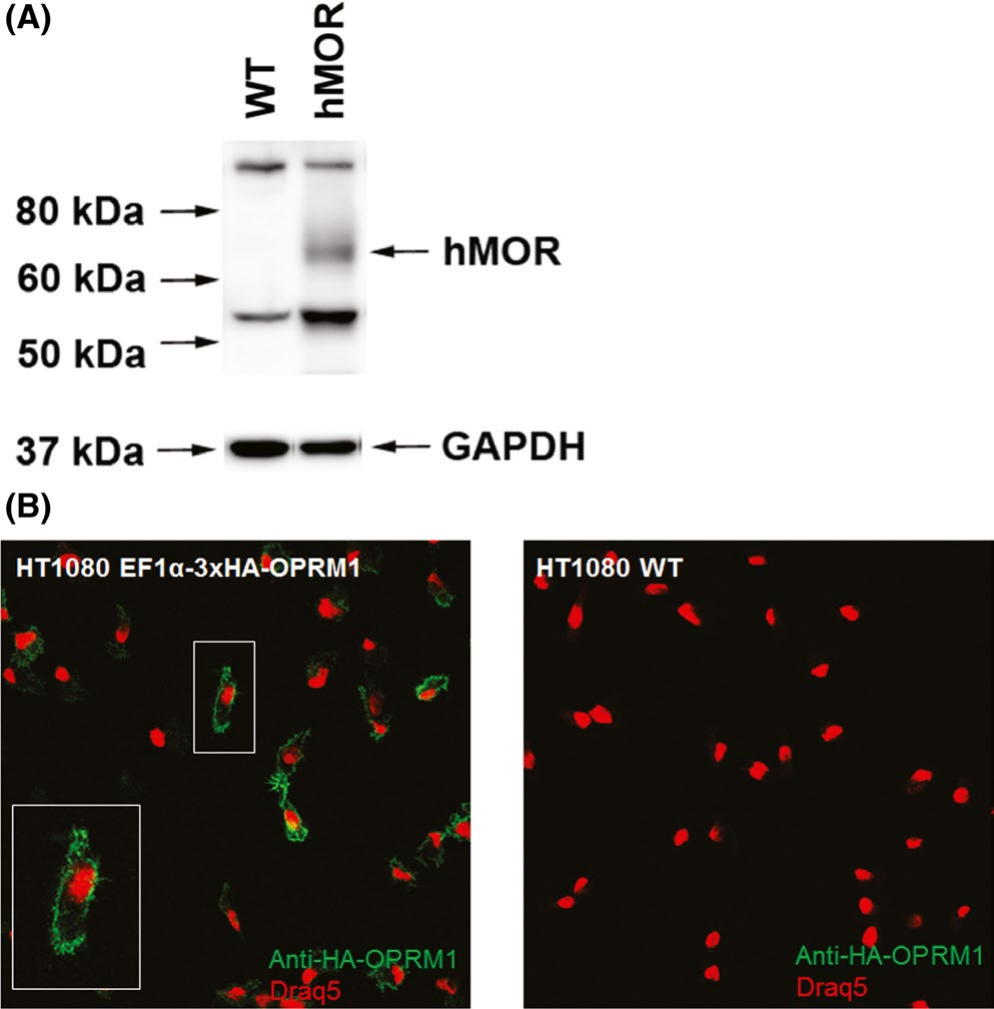
Validation of hMOR expression in HT1080 cells. (A) Western blot of HT1080 Wild Type (WT) and 3xHA‐OPRM1 lentivirus transduced cells as detected with an anti‐HA monoclonal antibody. (B) Confocal microscopy images detecting amino‐terminal 3x HA‐tagged hMOR using an anti‐HA primary antibody and Alexa488 conjugated secondary. Nucleus is counterstained with Draq5 and shown in red

A lentiviral construct (Figure S1) was used to generate stable transduced human 3x HA‐tagged OPRM1‐expressing HT1080 cells as described in Materials and Methods. Western blot was used to facilitate identification of antibodies suitable for immunohistochemistry and receptor internalization. The hMOR receptor was detected at the expected 65 kDa size in the hMOR but not in WT cells (Figure 3A). Confocal microscopy was also performed to further assess for hMOR expression at the subcellular level in the stable cell line. Human MOR expression was detected to be enriched at the cell membrane and in some endosomal compartments (Figure 3B).

### 3.3 Pharmacological validation of EF1α‐3xHA‐OPRM1‐expressing HT1080 cell (hMOR) line

**Figure 4:**
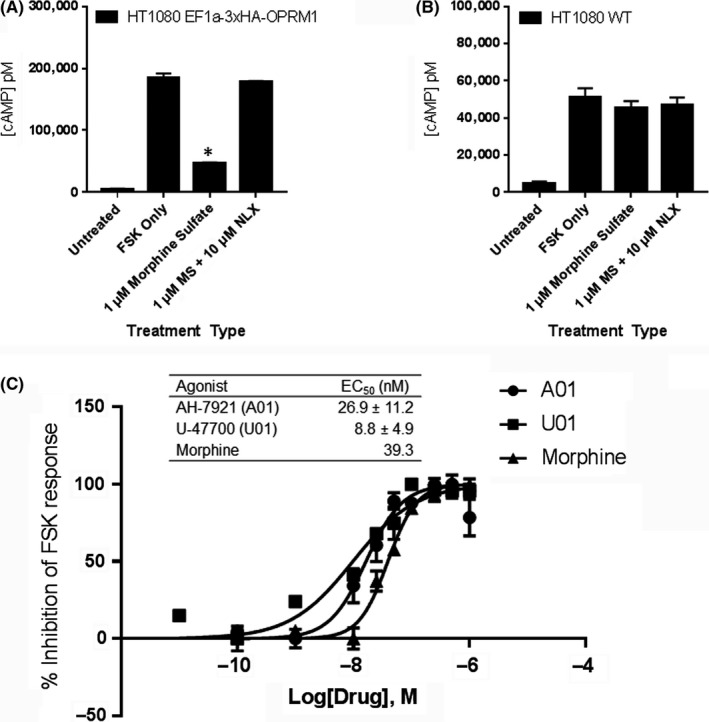
Pharmacological validation of HT1080 hMOR cells. (A) HT1080 hMOR cells are treated with 100 μmol L^−1^ of forskolin (FSK) only, FSK with 1 μmol L^−1^ morphine sulfate (MS), FSK with MS plus 10 μmol L^−1^ NLX, or PBS only (Untreated). (B) HT1080 WT is treated similarly. Data from representative experiment are shown. Statistical analysis is performed using one‐way ANOVA with Dunnett multiple comparison test with FSK only as standard. *P < 0.05. (C) Dose response curve and EC50 values table for morphine, AH‐7921 (A01), and U‐47700 (U01) in the HT1080 hMOR cell line. Each data point was performed in triplicate and repeated three times (n = 3). Data from representative experiment are shown

Human MOR‐expressing and WT cells were assessed for opiate‐mediated inhibition of FSK‐stimulated adenylyl cyclase activity to assess for hMOR Gα_i_ coupling in response to the prototypic opioid agonist MS to ensure that the 3x HA amino terminal tag did not interfere with receptor function. Treatment with MS (1 µmol L^−1^) resulted in a significant (*P* < 0.005) decrease in FSK‐induced cAMP accumulation in hMOR‐expressing cells but not in WT cells, validating surface expression and functional coupling of the epitope‐tagged hMOR in HT1080 cells and the lack of endogenous hMOR expression in WT HT1080 cells. Coadministration of MS with NLX completely inhibited the hMOR‐mediated Gα_i_ response and restored cAMP to FSK‐only levels (Figure 4).

Expanded MS dose ranges were assessed to determine an EC_50_ value for MS (39.3 nmol L^−1^), which is comparable to previously published EC_50_ values of MS in heterologous OPRM1‐expressing cells.^7^, ^8^, ^21^ The EC_50_ values for AH‐7921 (**A01**) and U‐47700 (**U01**) found to be 26.9 ± 11.2 nmol L^−1^ and 8.8 ± 4.9 nmol L^−1^, respectively, (Figure 4C) in hMOR‐expressing cells.

### 3.4 Potency of AH‐7921 and structural analogs at hMOR

**Figure 5:**
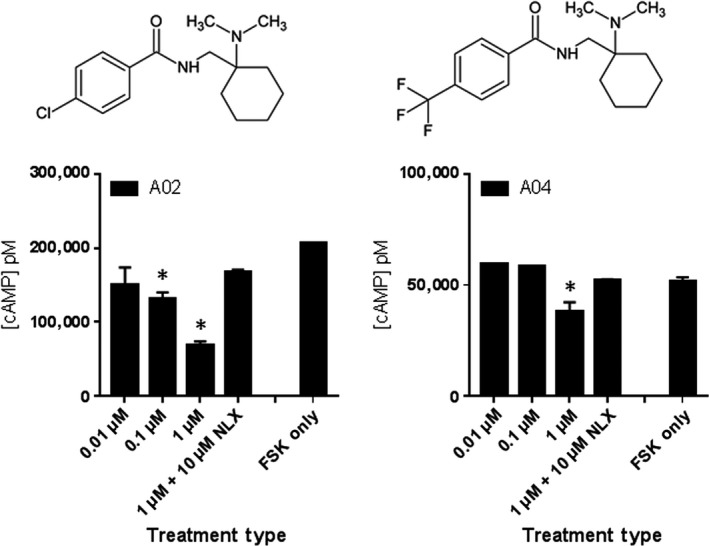
Drug potency of active AH‐7921 series compounds. HT1080 hMOR cells are treated with 100 μmol L^−1^ FSK only, or FSK with 0.01 μmol L^−1^ (low), 0.1 μmol L^−1^ (mid), 1 μmol L^−1^ (high) of the indicated compound, and high concentration dose of the compound with 10 μmol L^−1^ NLX. Each treatment dose was performed in triplicate (n = 2; data from representative experiment shown) and data were analyzed using one‐way ANOVA with Dunnett multiple comparison test and FSK only as standard. *P < 0.05

Seventeen analogs based on the AH‐7921 core structure were synthesized and assessed for hMOR pharmacological activity (Table S1). In addition to **A01** (AH‐7921), two analogs were found to result in significant suppression of FSK‐induced cAMP accumulation that was reversed by NLX coadministration (Figure 5). **A02** significantly decreased FSK‐induced cAMP levels at (0.1 µmol L^−1^) and showed further increasing dose‐dependent decrease of cAMP levels. Thus, of the AH‐series analogs we synthesized and tested, **A01** (Figure 4C; EC_50_ 26.9 ± 11.2 nmol L^−1^) and **A02** (Figure S6; EC_50_ 59.3 ± 2.0 nmol L^−1^) were classified as high potency hMOR agonists followed only by **A04,** which we classified as a low potency agonist (Figure 5). The remainder of the AH‐7921 compound series demonstrated either no activity or activity that was not reversible by NLX treatment (Table S1, Figure S2A).

### 3.5 Potency of U‐47700 and structural analogs at hMOR

**Figure 6:**
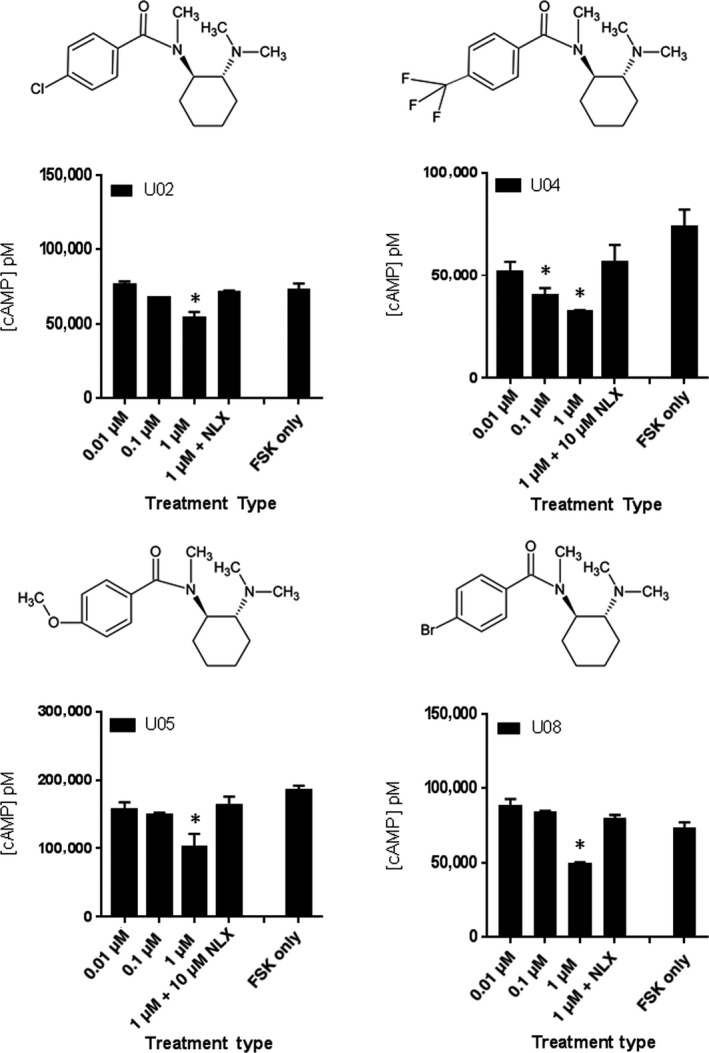
Drug potency of active U‐47700 series compounds. HT1080 hMOR cells are treated with 100 μmol L^−1^ FSK only, or FSK with 0.01 μmol L^−1^ (low), 0.1 μmol L^−1^ (mid), 1 μmol L^−1^ (high) of the indicated compound, and high concentration dose of the compound with 10 μmol L^−1^ NLX. Each treatment dose was performed in triplicate (n = 2; data from representative experiment shown) and data were analyzed using one‐way ANOVA with Dunnett multiple comparison test and FSK only as standard. *P < 0.05

Sixteen different U‐47700‐related analogs with (*R,R*) stereochemistry were synthesized and assessed for hMOR activity (Table S2). Of these, **U01** and **U04** (Figure 6) were classified as hMOR selective high potency agonists with EC_50_ values 8.8 ± 4.9 nmol L^−1^ and 26.0 ± 11.1 nmol L^−1^, respectively (Figure 4C, Figure S6). Pharmacological profiling also revealed **U02**, **U05,** and **U08** as low potency agonists. Analogous to findings with AH‐7921 analogs, most of the U‐47700 analogs were found to have no activity at hMOR at 1 μmol L^−1^ or lower concentrations. **U06**‐**07** and **U10**‐**17** were inactive based on comparison to FSK‐only control (Figure S3A). **U03** and **U09** showed significant decreases in cAMP levels in the range of concentrations tested, but their regulation of cAMP was not NLX reversible (Figure S3B). The summary of the U‐47700 series of opioid compound analogs is shown in Table S2.

### 3.6 Potency of Udes‐ and US‐series of analogs at hMOR

**Figure 7:**
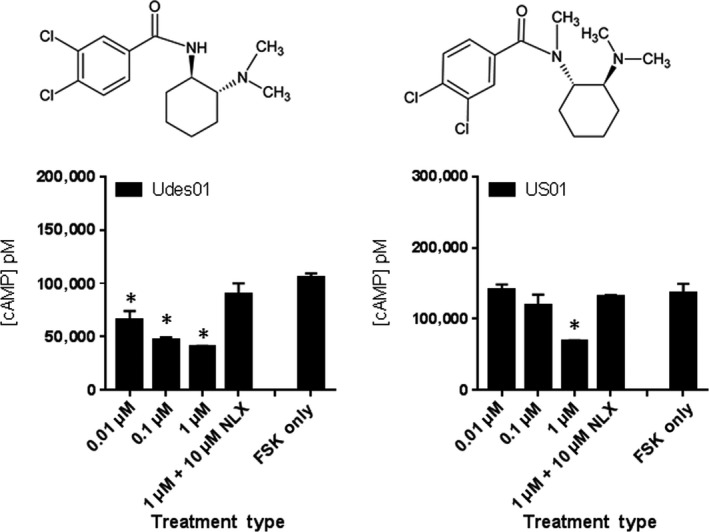
Drug potency of Udes01 and US01. HT1080 hMOR cells are treated with 100 μmol L^−1^ FSK only, or FSK with 0.01 μmol L^−1^ (low), 0.1 μmol L^−1^ (mid), 1 μmol L^−1^ (high) of the indicated compound, and high concentration dose of the compound with 10 μmol L^−1^ NLX. Each treatment dose was performed in triplicate (n = 2; data from representative experiment shown) and data were analyzed using one‐way ANOVA with Dunnett multiple comparison test and FSK only as standard. *P < 0.05

To study the effect of removing N‐methyl group on the amide in (*R*,*R*)‐U‐series, Udes‐series (compounds **Udes01**‐**09)** was synthesized and screened for hMOR signaling (Figure S4 and Table S3). Initial screening of **Udes01** resulted in a significant decrease of FSK‐induced cAMP levels at 10 nmol L^−1^, and specificity for hMOR was confirmed by NLX reversibility (Figure 7). The EC_50_ value of **Udes01** was subsequently determined to be 3.0 ± 0.3 nmol L^−1^ (Figure S6) characterizing it as the most potent analog that we discovered. The summary of the pharmacological activity of Udes‐series of compound analogs at the hMOR is shown in Table S3. The remaining Udes‐series (**Udes02**‐**09**) did not significantly alter FSK‐induced cAMP accumulation at any of the tested concentrations (Figure S4).

The US‐series was synthesized to assess for pharmacological activity of the *S*,*S* enantiomer of the U‐47700 analogs. The nine US‐series analogs were synthesized and screened for hMOR agonism (Table S4, Figure S5). Of these nine, only **US01** (3,4‐dichlorobenzoyl substituent) demonstrated significant decrease in cAMP levels at the highest screening dose of 1 μmol L^−1^ but not at the lower (0.1 or 0.01 µmol L^−1^) concentration ranges (Figure 7, Figure S5) indicating that the *S*,*S* enantiomer of U‐47700 was significantly less potent than the *R*,*R* enantiomer.

### 3.7 Efficacy of hMOR internalization by high potency AH‐7921, U‐47700 analogs

**Figure 8:**
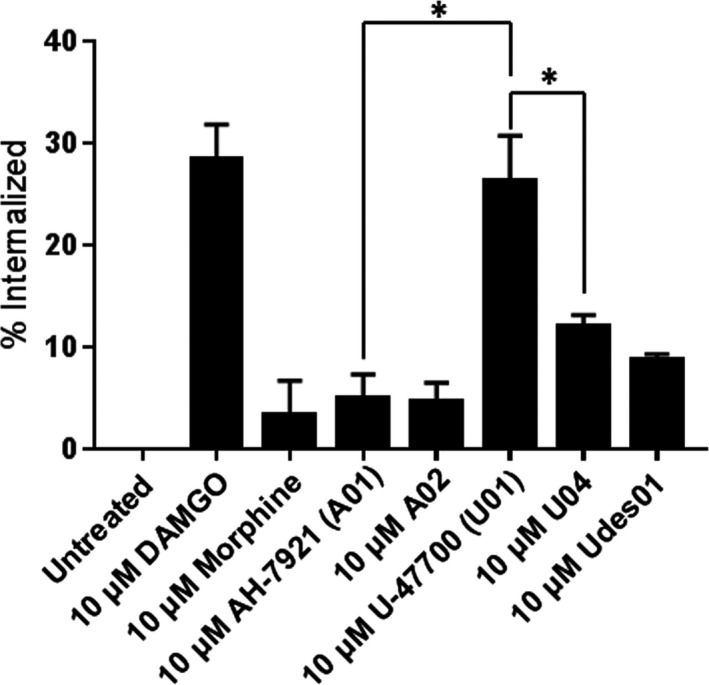
Measuring hMOR internalization levels after treatment with the high potency compounds. HT1080 hMOR cells were treated with the indicated concentration of drugs for 1 hour, then analyzed using flow cytometry to evaluate the changes in surface hMOR compared to untreated cells. The average internalization percentage is shown (n = 3). Data were analyzed using unpaired *t* test. *P < 0.05

Agonist‐induced receptor endocytosis remains a hallmark feature of GPCR activation and regulation and provides insights into the level of receptor desensitization that occurs after agonist stimulation.^8^ Thus, the AH‐7921, U‐47700, and Udes‐series compounds that demonstrated high potency were tested for their ability to induce hMOR internalization. The hMOR‐expressing cells were treated with saturating concentrations of 10 µmol L^−1^ [D‐Ala^2^,N‐MePhe^4^,Gly^5^‐ol] enkephalin (DAMGO) or 10 µmol L^−1^ morphine, which are reported to cause high levels or low levels of hMOR internalization, respectively.^8^, ^21^, ^22^ Similar results were found for DAMGO and morphine in our hMOR‐expressing cells (Figure 8). Interestingly, **A01** and **A02** (10 µmol L^−1^) resembled morphine treatment with only ~5% of cell‐surface receptors internalizing after 1 hour. In contrast, **U01** (10 µmol L^−1^) resulted in 6‐fold more hMOR internalization than morphine and **A01**, to levels similar to DAMGO (10 µmol L^−1^) treatment (~30% of cell‐surface receptors). **U04** and **Udes01** demonstrated hMOR internalization similar to morphine and the two AH‐series compounds (Figure 8).

## 4 DISCUSSION

We present a synthesis and screening strategy that enabled us to identify the pharmacological activity of over 50 ethylenediamine‐containing compounds at the hMOR. Of the high potency hMOR agonists that we found, the rank order of potencies for functional Gαi coupling and cAMP inhibition is as follows: **Udes01** > **U01** > **U04** = **A01 **≥ morphine sulfate > **A02**. We also classified **A04**, **U02**, **U05, U08,** and **US01** as low potency hMOR agonists. Our findings are somewhat aligned with findings detailed in the original patents and early studies, however, with some key differences. In the literature, compounds **A01** (AH‐7921), **A03**, and **A13** demonstrated 100% inhibition in the mouse hot plate test when dosed at 100 mg kg^−1^ sc, but only **A01** and **A03** demonstrated activity greater than that of aspirin in a phenylquinone test when orally dosed at 100 mg kg^−1^.^15^ The determination of ED_50_ (mg kg^−1^ sc) gave the following rank order or potency: **A01** 2.5 ≥ morphine 2.8 > **A02** 5.0 > **A15** 9.5 > **A13** 15.5.^23^ They also demonstrated that AH‐7921 had a high addictive liability in rats and rhesus monkeys. In our system, **A03, A13,** and **A15** failed to significantly suppress FSK‐induced cAMP accumulation based at 0.01, 0.1, or 1 μmol L^−1^ dosages in human OPRM1‐expressing cells (Figure S2) possibly reflecting differences in human and rodent OPRM1 receptor structures and underscoring the importance of validating SAR with human receptors.

In 1973, a group at Upjohn began looking at conformationally restrained analogs of phenampromide and by 1975 had developed the κ‐selective opioid analog, U‐50,488.^24^ Moving away from the N‐arylpropanamide structure, they developed the chemical series to which U‐47700 belongs. Rat tail flick (ED_50_ mg kg^−1^) and MOR‐related behavioral data (Straub tail, arched back, and increased locomotor activity) were reported.^25^, ^26^ A number of the compounds originally described are functionally characterized in this current study. The rank order of potency for analgesia (tail flick response time‐ED_50_ mg kg^−1^) is as follows: **U01** (U‐47700) 0.2 > **U04** 0.4 > **U02** 1.0 > morphine sulfate 1.5 > **Udes01** 11.^25^, ^26^ Each of these analogs demonstrated MOR selectivity based on NLX reversibility. Our rank order potency data for cAMP inhibition are similar to these trends showing **U01** to be the most potent U‐series analog followed by **U04**, then **U02**, **U05**, and **U08**; however, our data indicate that **Udes01** was ~3X more potent than **U01**, which is much more potent than indicated in the aforementioned studies. This could be explained by differences in human vs rodent MOR protein‐coding sequences or if the **Udes01** tested contained a racemic mixture, which is quite likely and could explain its much lower potency.

In light of this, our U‐series analog synthesis approach benefitted from the availability of the single stereoisomers of the advanced amine intermediates. This allowed for the synthesis of single stereoisomer analogs rather than the racemic versions of the analogs. Our findings, the (1*R*,2*R*) stereoisomer of U‐47700 (**U01**), were substantially more potent than the (1*S*,2*S*) stereoisomer (**US01)**. The influence of stereochemistry in the U‐series compounds on μ‐ vs κ‐opioid receptor selectivity has been reported^25^, ^27^ indicating that the (1*S*,2*S*) isomer has high κ‐opioid receptor selectivity, while the (1*R*,2*R*) isomer has high μ‐opioid receptor selectivity.

Differentiating the effects of single isomers vs racemic U‐series compounds may help delineate in vivo effects in humans mediated by μ‐ and κ‐opioid receptors. One report of a seized sample of U‐49900, a diethyl amine version of U‐47700 which is a dimethyl amine, demonstrated that it was a racemic mixture, as determined by circular dichroism.^28^ A study reporting the murine μ‐opioid receptor affinity and activity of U‐47700 provided by Cayman Chemicals, reports a binding affinity of 57 nmol L^−1^ for U‐47700 at the mouse μ‐opioid receptor vs 5 nmol L^−1^ for MS and an EC_50_ of 214 ± 23 nmol L^−1^ in a [^35^S]‐GTPγS binding assay,^29^ which is less potent than we found for (1*R*,2*R*)‐U‐47700 (**U01**). To date, Cayman Chemicals has confirmed that they supply racemic *trans* isomers of U‐47700 and not the single (1*R*,2*R*) stereoisomer (personal communication with Cayman Chemical customer service 12/10/2018).

It has been proposed that hMOR endocytosis is closely associated with the development of drug tolerance in the opiate user^8^, ^30^ and in mouse models.^14^ Our data reveal that **A01** and **A02** demonstrated low levels (~15%) of hMOR internalization with similar drug potency for Gα_i_ coupling. However, **U01** and **U04/Udes01** significantly (*P* < 0.05) diverged in their desensitization capacities: **U01** induced high levels of internalization (~26%) while **U04** and **Udes01** induced between 8% and 12% of cell surface hMORs to internalize. Thus, the levels of hMOR internalization for **A01**, **A02**, **U04**, and **Udes01** are similar to the reported internalization levels of well‐characterized opioids, such as morphine and fentanyl, while **U01** is much more similar to the endogenous opioids, the endorphins and the enkephalins.^7^, ^9^, ^31^ Thus, if the goal was to design new opiates with safer and improved pharmacodynamic properties that mimic endogenously produced “balanced/natural” opiate responses at the hMOR, further assessment of the (1*R*,2*R*) single isomer **U01** is likely to produce internalization and potentially receptor desensitization effects more aligned with endogenous opiates although further studies are needed.

In summary, our results demonstrate that this in vitro functional assay for hMOR pharmacology provides a foundation from which effects of these agents in humans can be anticipated and they provide a scaffold for the rational design of potentially superior analgesics that better mimic the endogenous opioid system.^14^, ^32^ Through our combinatorial approach, we discovered some novel moderately potent hMOR agonists. Whether these novel compounds have different tolerance profiles or addictive potential needs to be further investigated in vivo.

## DISCLOSURE

None to declare.

## AUTHOR CONTRIBUTIONS

ACZ, JLK, TH participated in research design; ACZ, JLK, TH, JRM, KY participated in writing and preparation of the manuscript, KY, VB, TL, TH, and JRM contributed to data acquisition.
