# Supplementary material for: Synthesis and pharmacological characterization of ethylenediamine synthetic opioids in human μ‐opiate receptor 1 (OPRM1) expressing cells
Source: Pharmacol Res Perspect. 2019 Aug 22;7(5):e00511. doi: 10.1002/prp2.511 (PMC6704399; doi:10.1002/prp2.511)
Supplement: Supplementary file 1 [file PRP2-7-e00511-s001.docx]

**SUPPORTING INFORMATION**

**Synthesis and pharmacological characterization of ethylenediamine synthetic opioids in human μ-opiate receptor 1 (OPRM1) expressing cells**^†^

Tom Hsu^1^, Jayapal Reddy Mallareddy^1^, Kayla Yoshida^1^, Vincent Bustamante^1^, Tim Lee^1^, John L. Krstenansky^1*^, Alexander C. Zambon^1*^

^1^Department of Biopharmaceutical Sciences, School of Pharmacy and Health Sciences, Keck Graduate Institute, Claremont, CA, USA

**Supplemental Tables**

**Supplemental Table 1.** Potency and specificity of AH-7921 and analogs in HT1080 EF1α-3xHA-OPRM1 cells.

| Substituent | Code *^a^* | Significant cAMP decre­­ase at: *^b^* | MOR specific? *^c^* (Naloxone reversible) | EC_50_ Value, nM *^d^* |
| --- | --- | --- | --- | --- |
| 3,4-dichlorobenzoyl * | **A01** | 0.1 µM | Yes | 26.9 ± 11.2 |
| 4-chlorobenzoyl * | **A02** | 0.1 µM | Yes | 59.3 ± 2.0 |
| 4-flurobenzoyl * | **A03** | No Effect | N/A | N/A |
| 4-trifluoromethylbenzoyl | **A04** | 1 µM | Yes | N/A |
| 4-methoxybenzoyl | **A05** | No Effect | No | N/A |
| 3,4-difluorobenzoyl | **A06** | No Effect | N/A | N/A |
| 3,4-dichlorobenzesulfonyl | **A07** | No Effect | N/A | N/A |
| 4-bromobenzoyl | **A08** | No Effect | N/A | N/A |
| 2-chloronicotinoyl | **A09** | No Effect | N/A | N/A |
| 1-naphthoyl | **A10** | No Effect | N/A | N/A |
| nicotinyl | **A11** | No Effect | N/A | N/A |
| isonicotinyl | **A12** | 1 µM | No | N/A |
| benzoyl * | **A13** | No Effect | N/A | N/A |
| 1-adamantanecarbonyl | **A14** | 0.1 µM | No | N/A |
| 3-chlorobenzoyl * | **A15** | No Effect | N/A | N/A |
| 3-fluorobenzoyl | **A16** | No Effect | N/A | N/A |
| 5-chlorothiophene-2-carboxylic acid | **A17** | No Effect | N/A | N/A |

*^a^* Numerical code name for the AH-7921 compound and its family of analogs.

*^b^* The lowest agonist concentration that demonstrated significant decrease in cAMP levels when compared with forskolin only control. No effect indicates no significant decrease in cAMP levels when tested at the highest dosage (1µM).

*^c^* Agonist specificity for MOR, as determined by naloxone reversibility.

*^d^* EC_50_ value is the average of three independent experiments, performed in triplicate.

* Demonstrated potency in literature[13, 14].

**Supplemental Table 2.** Potency and specificity of U-47700 and analogs HT1080 EF1α-3xHA-OPRM1 cells.

| Substituent | Code *^a^* | | Significant cAMP decrease at: *^b^* | | MOR specific? *^c^* (Naloxone reversible) | | EC_50_ Value, nM *^d^* | |  |
| --- | --- | --- | --- | --- | --- | --- | --- | --- | --- |
| 3,4-dichlorobenzoyl * | | **U01** | | 0.01 µM | | Yes | | 8.8 ± 4.9 | |
| 4-chlorobenzoyl * | | **U02** | | 1 µM | | Yes | | N/A | |
| 4-flurobenzoyl | | **U03** | | 0.01 µM | | No | | N/A | |
| 4-trifluoromethylbenzoyl * | | **U04** | | 0.1 µM | | Yes | | 26.0 ± 11.1 | |
| 4-methoxybenzoyl | | **U05** | | 1 µM | | Yes | | N/A | |
| 3,4-difluorobenzoyl | | **U06** | | No Effect | | N/A | | N/A | |
| 3,4-dichlorobenzesulfonyl | | **U07** | | No Effect | | N/A | | N/A | |
| 4-bromobenzoyl | | **U08** | | 1 µM | | Yes | | N/A | |
| 2-chloronicotinoyl | | **U09** | | 1 µM | | No | | N/A | |
| 1-naphthoyl | | **U10** | | No Effect | | N/A | | N/A | |
| nicotinyl | | **U11** | | No Effect | | N/A | | N/A | |
| isonicotinyl | | **U12** | | No Effect | | N/A | | N/A | |
| benzoyl | | **U13** | | No Effect | | N/A | | N/A | |
| 1-adamantanecarbonyl | | **U14** | | No Effect | | N/A | | N/A | |
| 3-chlorobenzoyl | | **U15** | | No Effect | | N/A | | N/A | |
| 3-fluorobenzoyl | | **U16** | | No Effect | | N/A | | N/A | |
| 5-chlorothiophene-2-carboxylic acid | | **U17** | | No Effect | | N/A | | N/A | |

*^a^* Numerical code name for the U-47700 compound and its family of analogs.

*^b^* The lowest agonist concentration that demonstrated significant decrease in cAMP levels when compared with forskolin only control. No effect indicates no significant decrease in cAMP levels when tested at the highest dosage (1µM).

*^c^* Agonist specificity for MOR, as determined by naloxone reversibility.

*^d^* EC_50_ value is the average of three independent experiments, performed in triplicate.

* Demonstrated potency in literature[15, 38].

**Supplemental Table 3.** Potency and specificity of Udes-series analogs in HT1080 EF1α-3xHA-OPRM1 cells.

| Substituent | Code *^a^* | | Significant cAMP decrease at: *^b^* | | MOR specific? *^c^* (Naloxone reversible) | | EC_50_ Value, nM *^d^* | |  |
| --- | --- | --- | --- | --- | --- | --- | --- | --- | --- |
| 3,4-dichlorobenzoyl * | | **Udes01** | | 0.01 µM | | Yes | | 3.0 ± 0.3 | |
| 4-chlorobenzoyl | | **Udes02** | | No Effect | | N/A | | N/A | |
| 4-flurobenzoyl | | **Udes03** | | No Effect | | N/A | | N/A | |
| 4-trifluoromethylbenzoyl | | **Udes04** | | No Effect | | N/A | | N/A | |
| 4-methoxybenzoyl | | **Udes05** | | No Effect | | N/A | | N/A | |
| 3,4-difluorobenzoyl | | **Udes06** | | No Effect | | N/A | | N/A | |
| 3,4-dichlorobenzesulfonyl | | **Udes07** | | No Effect | | N/A | | N/A | |
| 4-bromobenzoyl | | **Udes08** | | No Effect | | N/A | | N/A | |
| 2-chloronicotinoyl | | **Udes09** | | No Effect | | N/A | | N/A | |

*^a^* Numerical code name for the Udes compound and its family of analogs.

*^b^* The lowest agonist concentration that demonstrated significant decrease in cAMP levels when compared with forskolin only control. No effect indicates no significant decrease in cAMP levels when tested at the highest dosage (1µM).

*^c^* Agonist specificity for MOR, as determined by naloxone reversibility.

*^d^* EC_50_ value is the average of three independent experiments, performed in triplicate.

* Demonstrated potency in literature[38].

**Supplemental Table 4.** Potency and specificity of US-series analogs in HT1080 EF1α-3xHA-OPRM1 cells

| Substituent | Code *^a^* | Significant cAMP decrease at: *^b^* | MOR specific? *^c^* (Naloxone reversible) | EC_50_ Value |
| --- | --- | --- | --- | --- |
| 3,4-dichlorobenzoyl | **US01** | 1 µM | Yes | N/A |
| 4-chlorobenzoyl | **US02** | No Effect | N/A | N/A |
| 4-flurobenzoyl | **US03** | No Effect | N/A | N/A |
| 4-trifluoromethylbenzoyl | **US04** | No Effect | N/A | N/A |
| 4-methoxybenzoyl | **US05** | No Effect | N/A | N/A |
| 3,4-difluorobenzoyl | **US06** | No Effect | N/A | N/A |
| 3,4-dichlorobenzesulfonyl | **US07** | No Effect | N/A | N/A |
| 4-bromobenzoyl | **US08** | No Effect | N/A | N/A |
| 2-chloronicotinoyl | **US09** | No Effect | N/A | N/A |

*^a^* Numerical code name for the US compound and its family of analogs.

*^b^* The lowest agonist concentration that demonstrated significant decrease in cAMP levels when compared with forskolin only control. No effect indicates no significant decrease in cAMP levels when tested at the highest dosage (1µM).

*^c^* Agonist specificity for MOR, as determined by naloxone reversibility.

**Supplemental Figures**


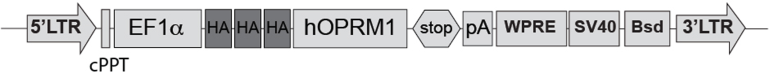


**Fig. S1. The EF1α-3xHA-OPRM1-2k7bsd lentiviral vector. Human *OPRM1* is epitope tagged with triple hemagglutinin (HA) tags at the N-terminus and is driven by the EF1α promoter.**

**
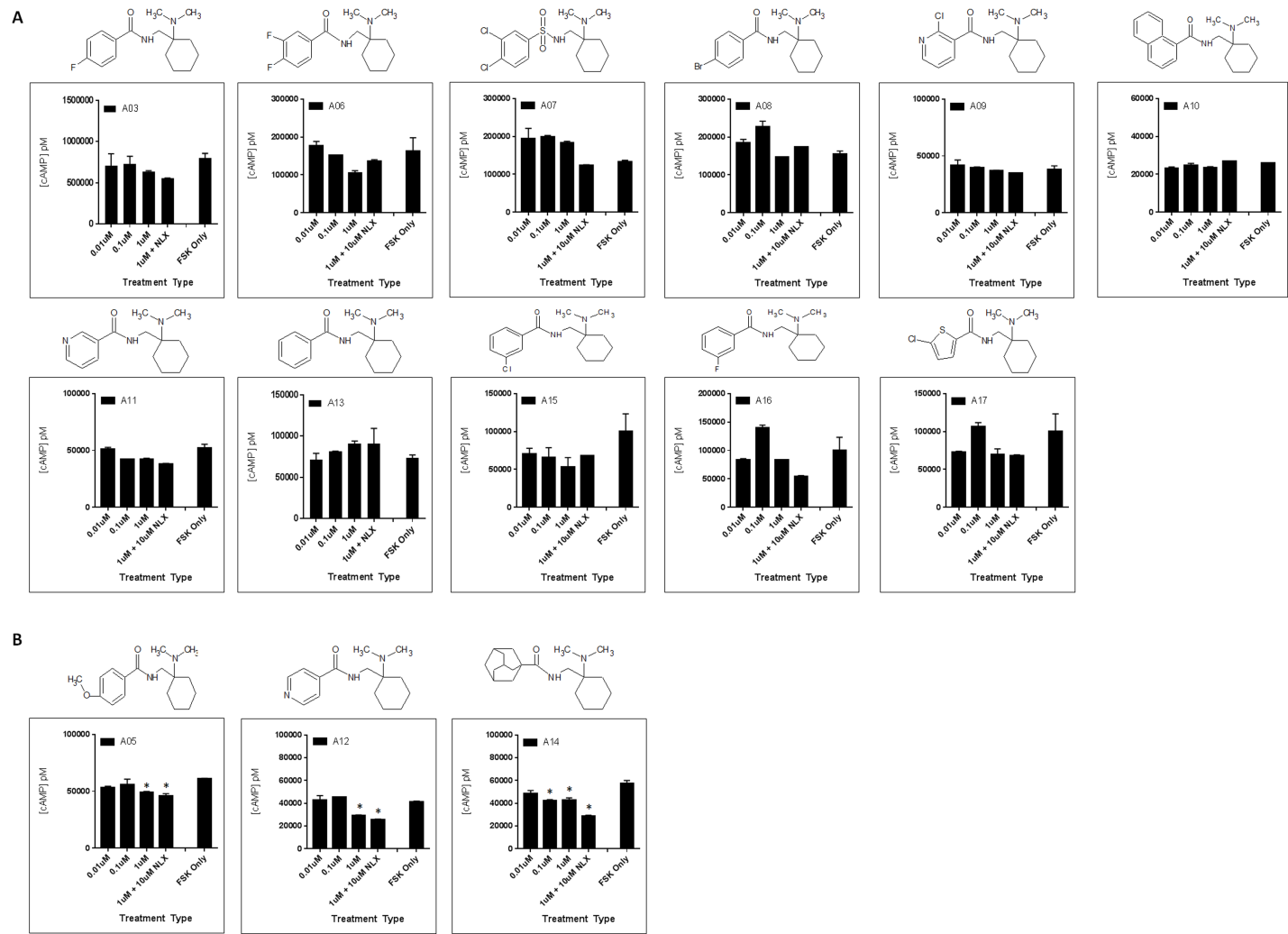
**

**Fig. S2. Drug potency of AH-7921 analogs. HT1080 EF1α-3xHA-OPRM1 cells are treated with 100µM FSK only, or FSK with 0.01µM (low), 0.1µM (mid), 1µM (high) of the indicated compound, and high concentration dose of the compound with 10µM NLX. Each treatment was performed in triplicate (n = 2). Data was analyzed using one-way ANOVA with Dunnett multiple comparison test and FSK only as standard. *p<0.05. (A) AH-7921 analogs that demonstrated no detectable drug activity. (B) AH-7921 analogs that demonstrated drug activity but the drug effects are not naloxone reversible.**


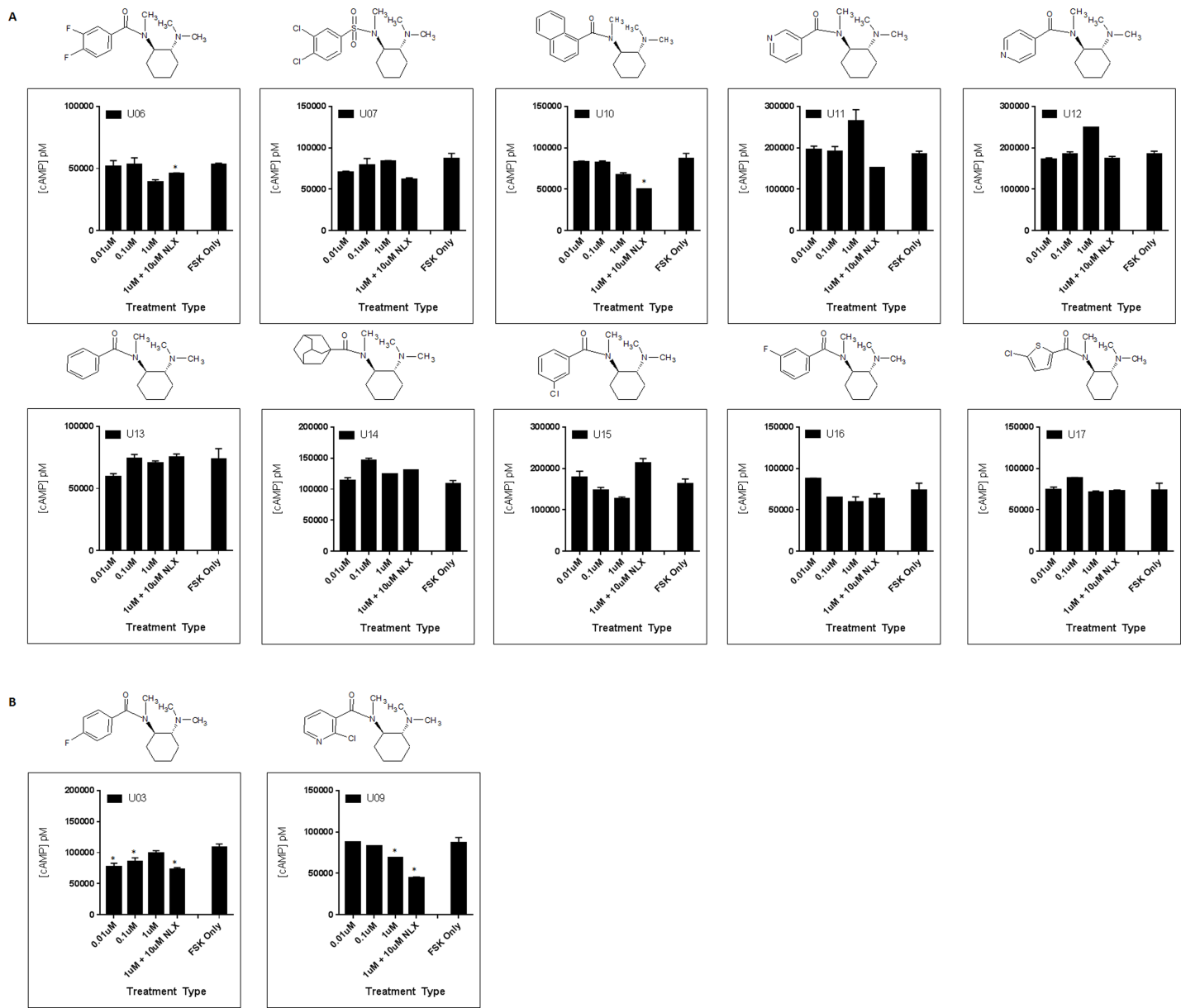


**Fig. S3. Drug potency of U-47700 analogs. HT1080 EF1α-3xHA-OPRM1 cells are treated with 100µM FSK only, or FSK with 0.01µM (low), 0.1µM (mid), 1µM (high) of the indicated compound, and high concentration dose of the compound with 10µM NLX. Each treatment was performed in triplicate (n = 2). Data was analyzed using one-way ANOVA with Dunnett multiple comparison test and FSK only as standard. *p<0.05. (A) U-47700 analogs that demonstrated no detectable drug activity. (B) U-47700 analogs that demonstrated drug activity but the drug effects are not naloxone reversible.**


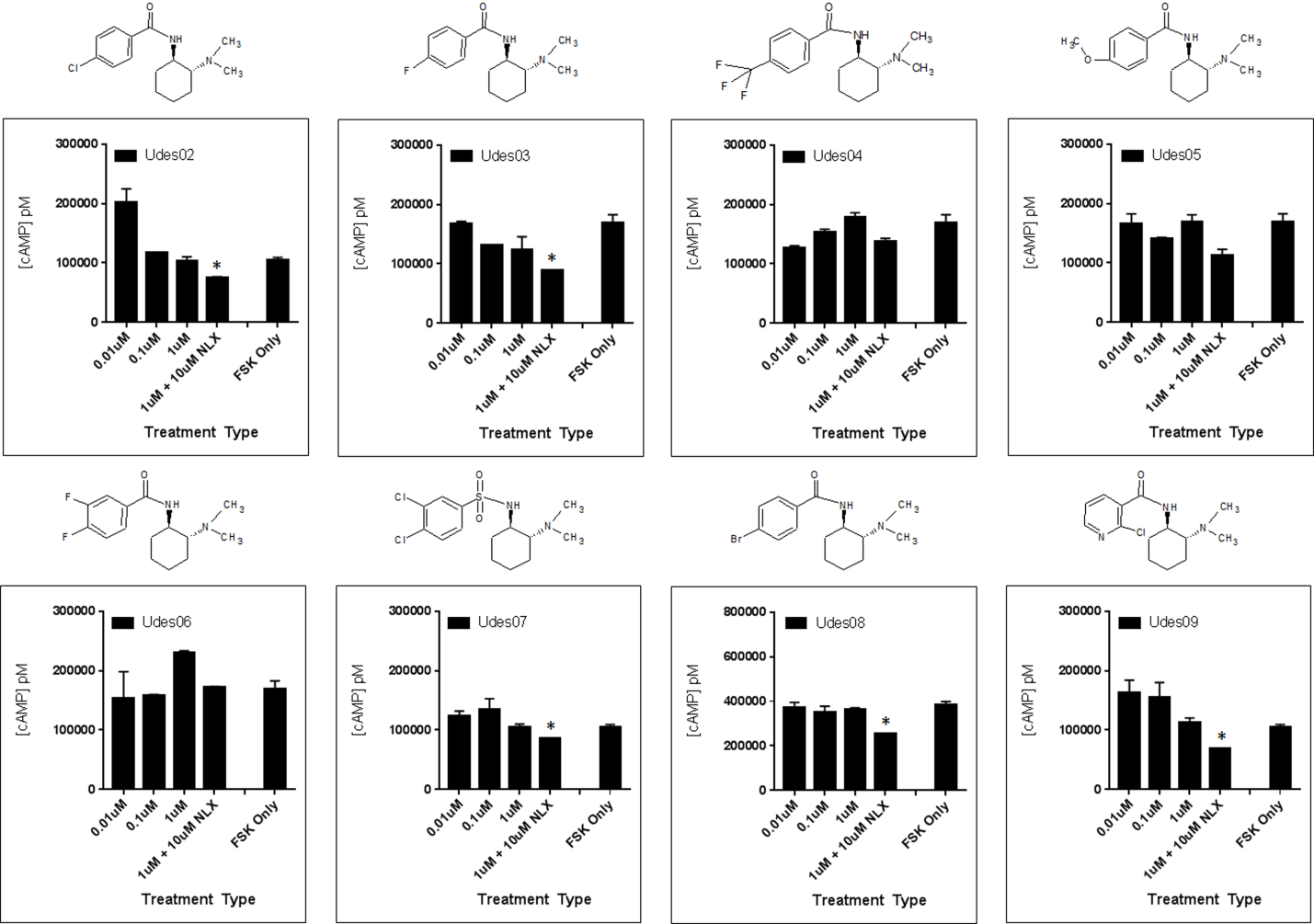


**Fig. S4. Drug potency of Udes-series analogs that demonstrated no detectable drug activity. HT1080 EF1α-3xHA-OPRM1 cells are treated with 100µM FSK only, or FSK with 0.01µM (low), 0.1µM (mid), 1µM (high) of the indicated compound, and high concentration dose of the compound with 10µM NLX. Each treatment was performed in triplicate (n = 2). Data was analyzed using one-way ANOVA with Dunnett multiple comparison test and FSK only as standard. *p<0.05.**


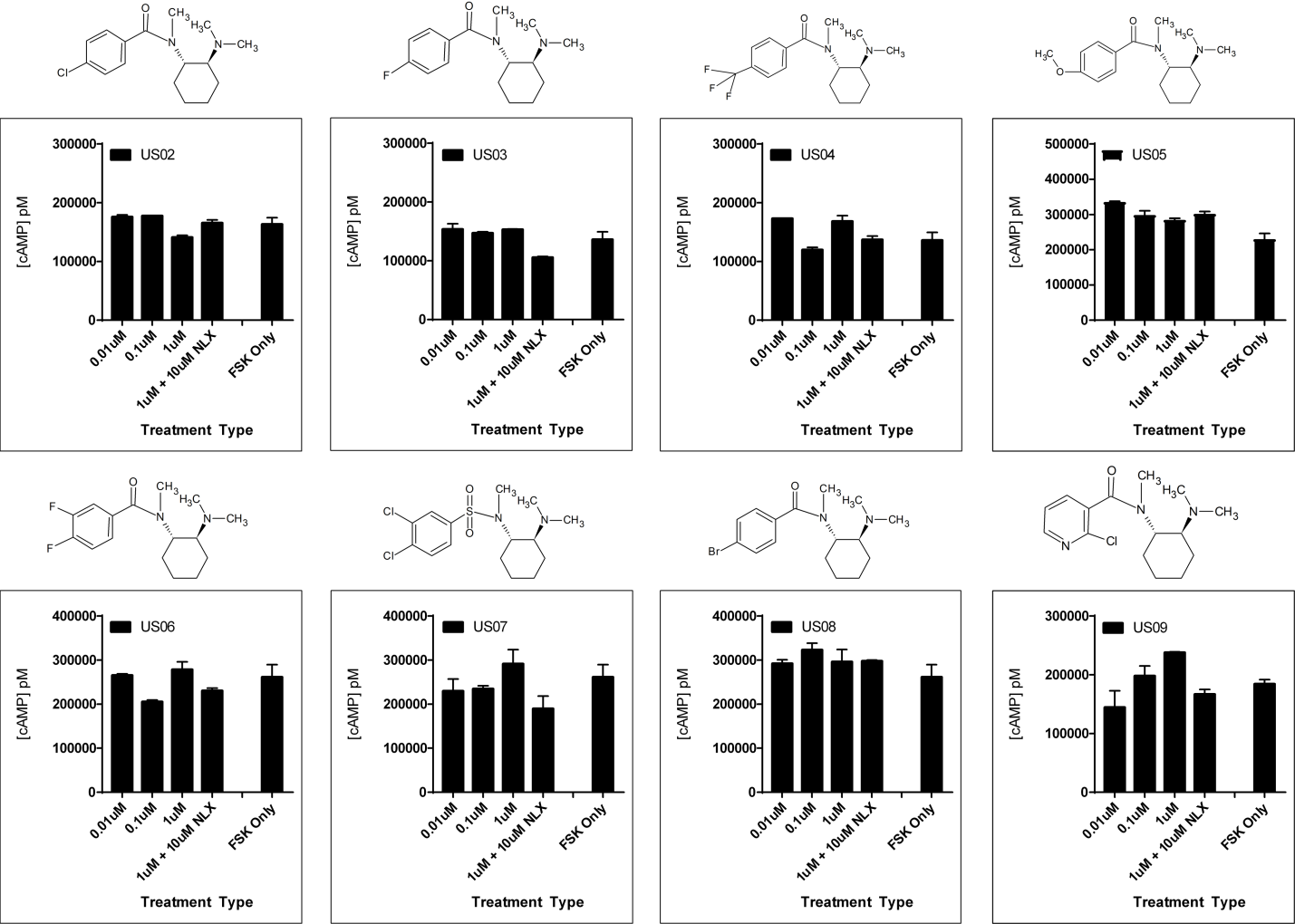


**Fig. S5. Drug potency of US-series analogs that demonstrated no detectable drug activity. HT1080 EF1α-3xHA-OPRM1 cells are treated with 100µM FSK only, or FSK with 0.01µM (low), 0.1µM (mid), 1µM (high) of the indicated compound, and high concentration dose of the compound with 10µM NLX. Each treatment was performed in triplicate (n = 2). Data was analyzed using one-way ANOVA with Dunnett multiple comparison test and FSK only as standard. *p<0.05.**


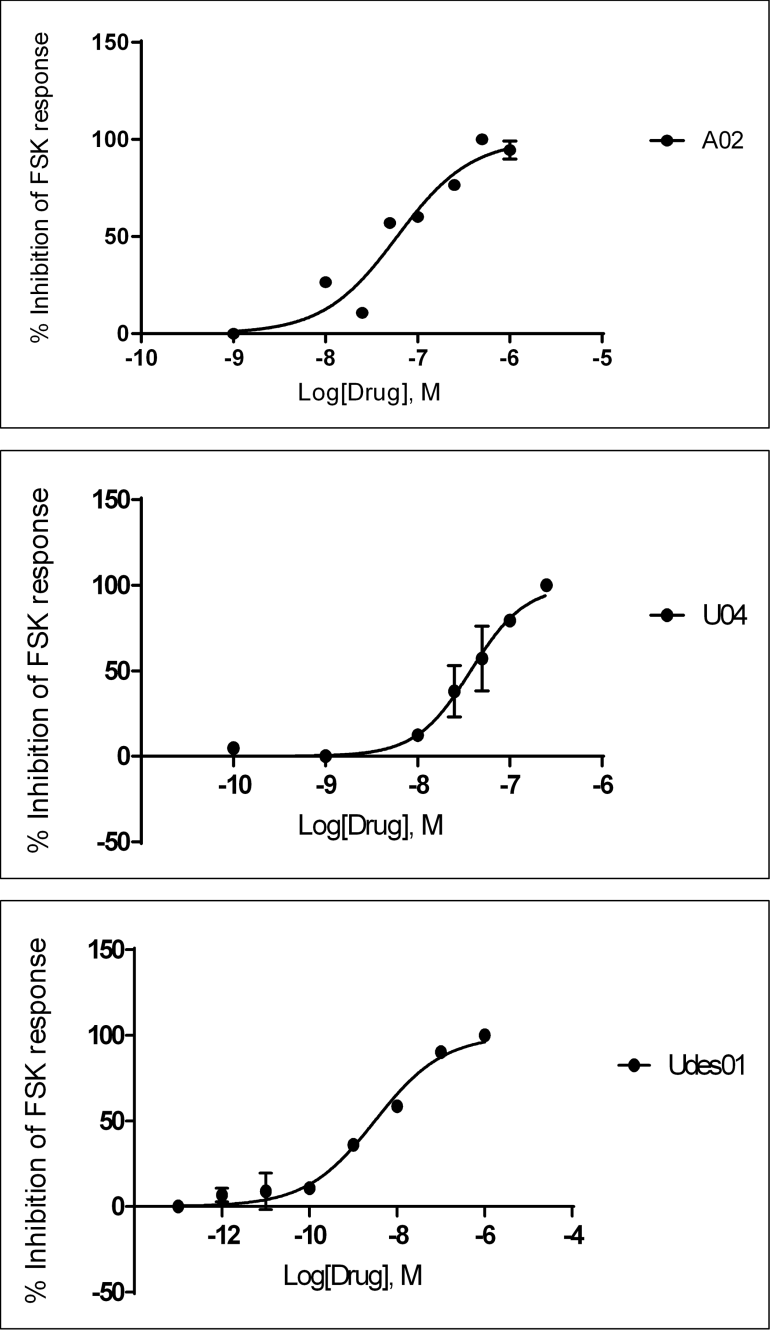


**Fig. S6. Dose response curves for the compounds that demonstrated high potency. Each data point was performed in triplicates(n = 3). Data from representative experiment is shown.**

**Compound Characterization**

2.4.1 **A01** (1-(3,4-dichlorobenzamidomethyl)-cyclohexyldimethylamine HCl)

^1^H NMR (400 MHz, METHANOL-*d*_4_) δ ppm 1.26 - 1.49 (m, 1 H), 1.59 - 1.85 (m, 7 H), 1.97 (br d, *J*=11.35 Hz, 2 H), 2.92 (s, 6 H), 3.88 (s, 2 H), 7.64 (d, *J*=8.61 Hz, 1 H), 7.84 (dd, *J*=8.22, 2.35 Hz, 1 H), 8.09 (d, *J*=1.96 Hz, 1 H). ^13^C NMR (101 MHz, METHANOL-*d*_4_) δ ppm 21.73, 24.18, 28.77, 37.30, 39.01, 68.04, 127.02, 129.82, 130.48, 132.73, 132.99, 136.30, 167.79. HRMS-ESI (+) calcd *m/z* for C_16_H_23_Cl_2_N_2_O^+^ 329.1187 (M+H)^+^, found 329.1230. GCMS 99.00% purity t­­_R_=16.99 min.

2.4.2 **A02** (1-(4-chlorobenzamidomethyl)-cyclohexyldimethylamine HCl)

^1^H NMR (400 MHz, METHANOL-*d*_4_) δ ppm 1.22 - 1.46 (m, 1 H) 1.57 - 1.85 (m, 7 H) 1.94 (br d, *J*=12.52 Hz, 2 H) 2.87 (s, 6 H) 3.85 (s, 2 H) 7.36 - 7.49 (m, 2 H) 7.85 - 7.97 (m, 2 H). HRMS-ESI (+) calcd *m/z* for C_16_H_24_ClN_2_O^+^ 295.1577 (M+H)^+^, found 295.1627. GCMS >99.00 % purity t­­_R_=15.97 min.

2.4.3. **A03** (1-(4-fluorobenzamidomethyl)-cyclohexyldimethylamine HCl)

^1^H NMR (400 MHz, CHLOROFORM-*d*) δ ppm 1.08 - 1.24 (m, 1 H), 1.44 - 1.63 (m, 3 H), 1.66 - 1.72 (m, 1 H), 1.73 - 1.88 (m, 3 H), 1.98 (br d, *J*=10.17 Hz, 2 H), 2.78 (d, *J*=5.09 Hz, 6 H), 4.03 (d, *J*=5.87 Hz, 2 H), 7.11 (t, *J*=8.05 Hz, 2 H), 8.17 - 8.35 (m, 2 H), 8.28 (s, 1 H), 11.96 (br s, 1 H). HRMS-ESI (+) calcd *m/z* for C_16_H_24_FN_2_O^+^ 279.1873 (M+H)^+^, found 279.1934. GCMS >99.00 % purity t­­_R_=14.65 min.

2.4.4. **A04** (1-(4-trifluoromethylbenzamidomethyl)-cyclohexyldimethylamine HCl)

^1^H NMR (400 MHz, CHLOROFORM-*d*) δ ppm 1.06 - 1.32 (m, 2 H), 1.52 - 1.67 (m, 3 H), 1.70 - 1.88 (m, 3 H), 1.99 (br d, *J*=10.56 Hz, 2 H), 2.79 (d, *J*=5.09 Hz, 6 H), 4.05 (d, *J*=5.48 Hz, 2 H), 7.71 (m, *J*=8.22 Hz, 2 H), 8.36 (m, *J*=8.22 Hz, 2 H), 9.03 (br s, 1 H), 11.99 (br s, 1 H). HRMS-ESI (+) calcd *m/z* for C_17_H_24_F_3_N_2_O^+^ 329.1841 (M+H)^+^, found 329.1906. GCMS 92.47 % purity t­­_R_=14.31 min.

2.4.5. **A05** (1-(4-methoxybenzamidomethyl)-cyclohexyldimethylamine HCl)

^1^H NMR (400 MHz, CHLOROFORM-*d*) δ ppm 1.09 - 1.23 (m, 1 H), 1.39 - 1.64 (m, 3 H), 1.67 - 1.74 (m, 1 H), 1.74 - 1.88 (m, 3 H), 1.99 (br d, *J*=10.56 Hz, 2 H), 2.78 (d, *J*=5.09 Hz, 6 H), 3.82 (s, 3 H), 4.02 (d, *J*=5.87 Hz, 2 H), 6.90 - 6.98 (m, 2 H), 8.11 - 8.24 (m, 2 H), 8.52 (br s, 1 H), 11.96 (br s, 1 H). HRMS-ESI (+) calcd *m/z* for C_17_H_27_N_2_O_2_^+^ 291.2073 (M+H)^+^, found 291.2140. GCMS 93.21 % purity t­­_R_=16.70 min.

2.4.6. **A06** (1-(3,4-difluorobenzamidomethyl)-cyclohexyldimethylamine HCl)

^1^H NMR (400 MHz, METHANOL-*d*_4_) δ ppm 1.23 – 1.47 (m, 1 H), 1.58 – 1.84 (m, 7 H), 1.94 (br d, *J*=11.74 Hz, 2 H), 2.89 (s, 6 H), 3.85 (s, 2 H), 7.31 – 7.40 (m, 1 H), 7.63 – 7.88 (m, 2 H). HRMS-ESI (+) calcd *m/z* for C_16_H_23_F_2_N_2_O^+^ 297.1778 (M+H)^+^, found 297.1844. GCMS 99.38 % purity t_R_=14.40 min.

2.4.7. **A07** (1-(3,4-dichlorobenzenesulfonamidomethyl)-cyclohexyldimethylamine HCl)

^1^H NMR (400 MHz, CHLOROFORM-*d*) δ ppm 1.05 - 1.31 (m, 1 H), 1.44 (q, *J*=12.39 Hz, 2 H), 1.59 - 1.70 (m, 2 H), 1.74 - 1.88 (m, 3 H), 2.00 (br d, *J*=12.13 Hz, 2 H), 2.84 (br d, *J*=4.70 Hz, 6 H), 3.28 (d, *J*=6.65 Hz, 2 H), 7.60 (d, *J*=8.61 Hz, 1 H), 7.84 - 8.03 (m, 2 H), 8.07 (d, *J*=1.96 Hz, 1 H), 8.20 (br t, *J*=6.65 Hz, 1 H), 10.75 (br s, 1 H). HRMS-ESI (+) calcd *m/z* for C_15_H_23_Cl_2_N_2_O_2_S^+^ 365.0857 (M+H)^+^, found 365.0878. GCMS 97.94 % purity t_R_=17.42 min.

2.4.8. **A08** (1-(4-bromobenzamidomethyl)-cyclohexyldimethylamine HCl)

^1^H NMR (400 MHz, CHLOROFORM-*d*) δ ppm 1.55 - 1.84 (m, 8 H), 1.98 (br d, *J*=10.17 Hz, 2 H), 2.78 (d, *J*=5.09 Hz, 6 H), 4.03 (d, *J*=5.87 Hz, 2 H), 7.58 (d, *J*=8.61 Hz, 2 H), 7.82 (d, *J*=8.61 Hz, 2 H), 8.85 (br s, 1 H), 12.00 (br s, 1 H). HRMS-ESI (+) calcd *m/z* for C_16_H_24_BrN_2_O^+^ 339.1072 (M+H)^+^, found 339.1112. GCMS 97.23 % purity t_R_=16.74 min.

2.4.9. **A09** (1-(2-chloronicotinamidomethyl)-cyclohexyldimethylamine HCl)

^1^H NMR (400 MHz, CHLOROFORM-*d*) δ ppm 1.21 (br s, 1 H), 1.54 (br s, 2 H), 1.59 - 1.67 (m, 3 H), 1.69 - 1.92 (m, 3 H), 2.02 (br d, *J*=8.22 Hz, 1 H), 2.77 (d, *J*=5.09 Hz, 6 H), 4.01 (d, *J*=5.87 Hz, 2 H), 7.31 (dd, *J*=7.63, 4.89 Hz, 1 H), 8.04 (dd, *J*=7.43, 1.96 Hz, 1 H), 8.43 (dd, *J*=4.70, 1.96 Hz, 1 H), 8.85 (br s, 1 H), 11.87 (br s, 1 H). HRMS-ESI (+) calcd *m/z* for C_15_H_23_ClN_3_O^+^ 296.1530 (M+H)^+^, found 296.1576. GCMS 99.23 % purity t_R_=15.93 min.

2.4.10. **A10** (1-(1-naphthamidomethyl)-cyclohexyldimethylamine HCl)

^1^H NMR (400 MHz, CHLOROFORM-*d*) δ ppm 1.10 - 1.32 (m, 1 H), 1.15 - 1.58 (m, 7 H), 1.92 (br d, *J*=12.13 Hz, 2 H), 2.75 (s, 6 H), 7.31 - 7.51 (m, 3 H), 7.68 (dd, *J*=7.04, 1.17 Hz, 1 H), 7.74 - 7.78 (m, 1 H), 7.83 (d, *J*=8.61 Hz, 1 H)), 8.06 - 8.22 (m, 1 H). HRMS-ESI (+) calcd *m/z* for C_20_H_27_N_2_O^+^ 311.2123 (M+H)^+^, found 311.2193. GCMS 96.83 % purity t_R_=18.27 min.

2.4.11. **A11** (1-(nicotinamidomethyl)-cyclohexyldimethylamine HCl)

^1^H NMR (400 MHz, METHANOL-*d*_4_) δ ppm 1.29 (qd, *J*=12.33, 3.72 Hz, 1 H), 1.61 - 1.87 (m, 7 H), 1.98 (br d, *J*=11.74 Hz, 2 H), 2.89 (s, 6 H), 3.99 (s, 2 H), 8.08 - 8.17 (m, 1 H), 8.92 (dd, *J*=5.87, 0.78 Hz, 1 H), 9.20 (dt, *J*=8.12, 1.61 Hz, 1 H), 9.55 (d, *J*=1.96 Hz, 1 H). HRMS-ESI (+) calcd *m/z* for C_15_H_24_N_3_O^+^ 262.1919 (M+H)^+^, found 262.1969. GCMS 98.19 % purity t_R_=15.11 min.

2.4.12. **A12** (1-(isonicotinamidomethyl)-cyclohexyldimethylamine HCl)

^1^H NMR (400 MHz, CHLOROFORM-*d*) δ ppm 1.19 - 1.36 (m, 1 H), 1.60 - 1.67 (m, 1 H), 1.70 - 1.92 (m, 6 H), 2.00 (br d, *J*=11.74 Hz, 2 H), 2.88 - 2.92 (m, 6 H), 4.04 (s, 2 H), 8.76 (d, *J*=5.88 Hz, 2 H), 8.85 - 9.07 (m, 2 H). HRMS-ESI (+) calcd *m/z* for C_15_H_24_N_3_O^+^ 262.1919 (M+H)^+^, found 262.1955. GCMS 91.78 % purity t_R_=15.09 min.

2.4.13. **A13** (1-(benzamidomethyl)-cyclohexyldimethylamine HCl)

^1^H NMR (400 MHz, METHANOL-*d*_4_) δ ppm 1.26 – 1.50 (m, 1 H), 1.60 – 1.85 (m, 7 H), 1.97 (br d, *J*=12.13 Hz, 2 H), 2.92 (s, 6 H), 3.86 (s, 2 H), 7.42 – 7.59 (m, 3 H), 7.89 (d, *J*=7.66 Hz, 2 H). HRMS-ESI (+) calcd *m/z* for C_16_H_25_N_2_O^+^ 261.1967 (M+H)^+^, found 261.2032. GCMS 98.76 % purity t_R_=14.82 min.

2.4.14. **A14** (1-(1-adamantanecarboxamidomethyl)-cyclohexyldimethylamine HCl)

^1^H NMR (400 MHz, CHLOROFORM-*d*) δ ppm 1.06 - 1.24 (m, 2 H), 1.44 - 1.68 (m, 6 H), 1.72 - 1.90 (m, 8 H), 1.94 (d, *J*=2.35 Hz, 6 H), 2.02 (br s, 3 H), 2.71 (d, *J*=5.09 Hz, 6 H), 3.78 (d, *J*=5.87 Hz, 2 H), 7.61 (br s, 1 H), 11.84 (br s, 1 H). HRMS-ESI (+) calcd *m/z* for C_20_H_35_N_2_O^+^ 319.2749 (M+H)^+^, found 319.2829. GCMS 99.22 % purity t_R_=17.27 min.

2.4.15. **A15** (1-(3-chlorobenzamidomethyl)-cyclohexyldimethylamine HCl)

^1^H NMR (400 MHz, METHANOL-*d*_4_) δ ppm 1.33 (q, *J*=11.48 Hz, 1 H), 1.59 - 1.85 (m, 7 H), 1.96 (br d, *J*=12.13 Hz, 2 H), 2.91 (s, 6 H), 3.86 (s, 2 H), 7.46 (t, *J*=8.02 Hz, 1 H), 7.56 (d, *J*=8.02 Hz, 1 H), 7.71 - 7.87 (m, 1 H), 7.92 (s, 1 H). HRMS-ESI (+) calcd *m/z* for C_16_H_24_ClN_2_O^+^ 295.1577 (M+H)^+^, found 295.1634. GCMS 95.84 % purity t_R_=15.85 min.

2.4.16. **A16** (1-(3-fluorobenzamidomethyl)-cyclohexyldimethylamine HCl)

^1^H NMR (400 MHz, METHANOL-*d*_4_) δ ppm 1.22 - 1.49 (m, 1 H), 1.60 - 1.85 (m, 7 H), 1.96 (br d, *J*=12.13 Hz, 2 H), 2.91 (s, 6 H), 3.86 (s, 2 H), 7.29 (td, *J*=8.41, 2.74 Hz, 1 H), 7.40 - 7.56 (m, 1 H), 7.64 (br d, *J*=9.39 Hz, 1 H), 7.72 (d, *J*=7.43 Hz, 1 H). HRMS-ESI (+) calcd *m/z* for C_16_H_24_FN_2_O^+^ 279.1873 (M+H)^+^, found 279.1938. GCMS 95.78 % purity t_R_=14.53 min.

2.4.17. **A17** (1-(5-chlorothienyl-2-carboxamidomethyl)-cyclohexyldimethylamine HCl)

^1^H NMR (400 MHz, METHANOL-*d*_4_) δ ppm 1.15 – 1.41 (m, 1 H), 1.58 – 1.85 (m, 8 H), 1.90 – 1.99 (m, 1 H), 2.79 – 2.94 (m, 6 H), 3.83 (s, 2 H), 7.02 (d, *J*=4.30 Hz, 1 H), 7.72 (d, *J*=4.30 Hz, 1 H). HRMS-ESI (+) calcd *m/z* for C_14_H_22_ClN_2_OS^+^ 301.1141 (M+H)^+^, found 301.1176. GCMS 96.83 % purity t_R_=15.70 min.

2.4.18. **U01** (N-((*1R,2R*)-2-(dimethylamino)cyclohexyl)-3,4-dichloro-N-methylbenzamide HCl)

^1^H NMR (400 MHz, METHANOL-*d*_4_) δ ppm 1.35 – 1.56 (m, 2 H), 1.64 (q, *J*=12.13 Hz, 1 H), 1.77 – 2.02 (m, 4 H), 2.08 – 2.34 (m, 1 H), 2.88 (s, 3 H), 2.93 (s, 3H), 2.94 (s, 3 H), 3.71 (td, *J*=11.54, 3.52 Hz, 1 H), 4.53 – 4.80 (m, 1 H) 7.48 – 7.66 (m, 2 H), 7.73 – 7.89 (m, 1 H). ^13^C NMR (101 MHz, METHANOL-*d*_4_) δ ppm 22.77, 23.62, 24.22, 28.86, 32.10, 36.47, 42.27, 53.65, 63.74, 126.97, 129.56, 130.25, 132.21, 133.87, 135.88, 171.64. HRMS-ESI (+) calcd *m/z* for C_16_H_23_Cl_2_N_2_O^+^ 329.1187 (M+H)^+^, found 329.1220. GCMS 99.24 % purity t_R_=16.01 min.

2.4.19. **U02** (N-((*1R,2R*)-2-(dimethylamino)cyclohexyl)-4-chloro-N-methylbenzamide HCl)

^1^H NMR (400 MHz, METHANOL-*d*_4_) δ ppm 1.33 – 1.56 (m, 2 H), 1.56 – 1.72 (m, 1 H), 1.77 – 2.04 (m, 4 H), 2.23 (br d, *J*=10.96 Hz, 1 H), 2.90 (s, 3 H), 2.94 (s, 3 H), H), 3.69 (td, *J*=11.54, 3.52 Hz, 1 H), 4.73 (m, 1 H), 7.46 (d, *J*=8.22 Hz, 2 H), 7.57 (d, *J*=8.22 Hz, 2 H). HRMS-ESI (+) calcd *m/z* for C_16_H_24_ClN_2_O^+^ 295.1577 (M+H)^+^, found 295.1613. GCMS 98.91 % purity t_R_=14.02 min.

2.4.20. **U03** (N-((*1R,2R*)-2-(dimethylamino)cyclohexyl)-4-fluoro-N-methylbenzamide)

^1^H NMR (400 MHz, CHLOROFORM-*d*) δ ppm 0.75 - 0.96 (m, 1 H), 1.06 - 1.27 (m, 2 H), 1.32 - 1.56 (m, 1 H), 1.73 - 1.92 (m, 3 H), 2.00 (s, 2 H), 2.24 (s, 4 H), 2.32 - 2.54 (m, 1.5 H), 2.74 (s, 2 H), 2.89 (s, 1.5 H) 3.30 (td, *J*=10.96, 3.91 Hz, 1 H), 4.44 - 4.63 (m, 1 H), 7.03 (td, *J*=8.61, 2.74 Hz, 2 H), 7.37 (ddd, *J*=8.51, 5.58, 2.35 Hz, 2 H). HRMS-ESI (+) calcd *m/z* for C_16_H_24_FN_2_O^+^ 279.1873 (M+H)^+^, found 279.1896. GCMS 98.75 % purity t_R_=13.38 min.

2.4.21. **U04** (N-((*1R,2R*)-2-(dimethylamino)cyclohexyl)-4-trifluoromethyl-N-methylbenzamide)

^1^H NMR (400 MHz, CHLOROFORM-*d*) δ ppm 1.00 - 1.26 (m, 2 H), 1.34 - 1.58 (m, 2 H), 1.70 - 1.92 (m, 4 H), 1.96 - 2.06 (m, 2 H), 2.26 (s, 3 H), 2.38 - 2.54 (m, 1 H), 2.71 (s, 2 H), 2.93 (s, 1 H) 3.22 (td, *J*=10.96, 3.91 Hz, 1 H) 4.57 (m, 1 H) 7.48 (t, *J*=7.83 Hz, 2 H), 7.62 (br d, *J*=8.22 Hz, 2 H). HRMS-ESI (+) calcd *m/z* for C_17_H_24_F_3_N_2_O^+^ 329.1841 (M+H)^+^, found 329.1865. GCMS 99.17 % purity t_R_=13.20 min.

2.4.22. **U05** (N-((*1R,2R*)-2-(dimethylamino)cyclohexyl)-4-methoxy-N-methylbenzamide HCl)

^1^H NMR (400 MHz, METHANOL-*d*_4_) δ ppm 1.33 – 1.53 (m, 2 H), 1.62 (br s, 1 H), 1.73 – 1.92 (m, 2 H), 1.92 – 2.08 (m, 2 H), 2.21 (br d, *J*=11.74 Hz, 1 H), 2.90 (s, 3 H), 2.93 (s, 3 H), 2.98 (s, 3 H), 3.57 – 3.77 (m, 1 H), 3.84 (s, 3 H) 4.69 (m, 1 H), 6.98 (d, *J*=9.00 Hz, 2 H), 7.52 (br d, *J*=7.83 Hz, 2 H). HRMS-ESI (+) calcd *m/z* for C_17_H_27_N_2_O_2_^+^ 291.2073 (M+H)^+^, found 291.2131. GCMS >99.00 % purity t_R_=15.48 min.

2.4.23. **U06** (N-((*1R,2R*)-2-(dimethylamino)cyclohexyl)-3,4-difluoro-N-methylbenzamide HCl)

^1^H NMR (400 MHz, METHANOL-*d*_4_) δ ppm 1.31 – 1.56 (m, 2 H), 1.56 – 1.72 (m, 1 H), 1.78 – 2.03 (m, 4 H), 2.14 – 2.33 (m, 1 H), 2.89 (s, 3 H), 2.94 (s, 6 H) 3.57 – 3.77 (m, 1 H), 4.53 – 4.80 (m, 1 H), 7.25 – 7.50 (m, 2 H), 7.60 (t, *J*=8.80 Hz, 1 H). HRMS-ESI (+) calcd *m/z* for C_16_H_23_F_2_N_2_O^+^ 297.1778 (M+H)^+^, found 297.1830. GCMS 97.10 % purity t_R_=13.38 min.

2.4.24. **U07** (N-((*1R,2R*)-2-(dimethylamino)cyclohexyl)-3,4-dichloro-N-methylbenzsulfonamide)

^1^H NMR (400 MHz, METHANOL-*d*_4_) δ ppm 0.96 (br d, *J*=11.35 Hz, 1 H), 1.16 - 1.42 (m, 3 H), 1.49 - 1.72 (m, 2 H) 1.74 - 1.92 (m, 1 H), 2.07 - 2.33 (m, 1 H), 2.82 (s, 3 H), 2.90 (s, 3 H), 2.94 (s, 3H), 3.46 (td, *J*=11.44, 3.72 Hz, 1 H), 4.02 (td, *J*=10.76, 3.52 Hz, 1 H), 7.63 - 7.85 (m, 2 H), 8.05 (d, *J*=1.96 Hz, 1 H). HRMS-ESI (+) calcd *m/z* for C_15_H_23_Cl_2_N_2_O_2_S^+^ 365.0857 (M+H)^+^, found 365.0894. GCMS >99.00 % purity t_R_=16.69 min.

2.4.25. **U08** (N-((*1R,2R*)-2-(dimethylamino)cyclohexyl)-4-bromo-N-methylbenzamide)

^1^H NMR (400 MHz, METHANOL-*d*_4_) δ ppm 1.04 - 1.30 (m, 2 H), 1.58 - 1.85 (m, 3 H), 1.88 - 1.99 (m, 1 H), 2.04 (s, 2 H), 2.28 (s, 3 H), 2.50 - 2.71 (m, 1 H), 2.78 (s, 2 H) 2.87 - 2.97 (m, 1 H), 4.52 (br s, 1 H), 7.19 - 7.41 (m, 2 H), 7.46 - 7.71 (m, 2 H). HRMS-ESI (+) calcd *m/z* for C_16_H_24_BrN_2_O^+^ 339.1072 (M+H)^+^, found 339.1083. GCMS >99.00 % purity t_R_=15.65 min.

2.4.26. **U09** (N-((*1R,2R*)-2-(dimethylamino)cyclohexyl)-2-chloro-N-methylnicotinamide HCl)

^1^H NMR (400 MHz, METHANOL-*d*_4_) δ ppm 1.32 - 1.58 (m, 2 H), 1.59 - 1.76 (m, 1 H), 1.82 (m, 1 H), 1.85 - 2.09 (m, 3 H), 2.12 - 2.34 (m, 1 H), 2.86 (s, 3 H), 2.89 (s, 3 H), 2.94 (s, 3 H), 3.66 (m, 1 H), 4.90 (m, 1 H), 7.44 - 7.52 (m, 1 H) 8.32 (br s, 1 H), 8.44 (dd, *J*=5.09, 1.96 Hz, 1 H). HRMS-ESI (+) calcd *m/z* for C_15_H_23_ClN_3_O^+^ 296.1530 (M+H)^+^, found 296.1565. GCMS >99.00 % purity t_R_=15.21 min.

2.4.27. **U10** (N-((*1R,2R*)-2-(dimethylamino)cyclohexyl)-N-methyl-1-naphthylcarboxamide)

^1^H NMR (400 MHz, CHLOROFORM-*d*) δ ppm 1.15 - 1.38 (m, 2 H), 1.41 - 1.60 (m, 2 H), 1.76 - 1.91 (m, 3 H), 1.95 (br d, *J*=12.13 Hz, 1 H), 2.36 (s, 3 H) 2.41 (s, 3 H), 2.60 (s, 3 H) 2.97 - 3.18 (m, 1 H), 4.88 (m, 1 H), 7.46 (br t, *J*=7.24 Hz, 4 H), 7.77 - 7.85 (m, 2 H), 8.27 (br s, 1 H). HRMS-ESI (+) calcd *m/z* for C_20_H_27_N_2_O^+^ 311.2123 (M+H)^+^, found 311.2146. GCMS 97.11 % purity t_R_=17.05 min.

2.4.28. **U11** (N-((*1R,2R*)-2-(dimethylamino)cyclohexyl)-N-methyl-3-pyridinecarboxamide HCl)

^1^H NMR (400 MHz, METHANOL-*d*_4_) δ ppm 1.34 - 1.58 (m, 2 H), 1.58 - 1.74 (m, 1 H), 1.79 - 2.06 (m, 4 H), 2.13 - 2.31 (m, 1 H), 2.89 (s, 3 H), 2.93 (s, 3 H), 2.99 (s, 3 H), 3.59 - 3.80 (m, 1 H), 4.59 - 4.80 (m, 1 H), 7.88 (dd, *J*=7.83, 5.48 Hz, 1 H), 8.58 (br d, *J*=8.22 Hz, 1 H), 8.79 (dd, *J*=5.48, 1.57 Hz, 1 H), 9.03 - 9.15 (m, 1 H). HRMS-ESI (+) calcd *m/z* for C_15_H_24_N_3_O^+^ 262.1919 (M+H)^+^, found 262.1930. GCMS 97.72 % purity t_R_=14.22 min.

2.4.29. **U12** (N-((*1R,2R*)-2-(dimethylamino)cyclohexyl)-N-methyl-4-pyridinecarboxamide HCl)

^1^H NMR (400 MHz, METHANOL-*d*_4_) δ ppm 1.34 - 1.56 (m, 2 H), 1.58 - 1.73 (m, 1 H), 1.78 - 2.03 (m, 4 H), 2.20 - 2.33 (m, 1 H), 2.89 (s, 3 H), 2.91 (s, 3 H), 2.94 (s, 3 H), 3.70 (td, *J*=11.54, 3.13 Hz, 1 H), 4.71 - 4.79 (m, 1 H), 7.71 (d, *J*=5.09 Hz, 2 H), 8.67 (d, *J*=5.09 Hz, 2 H). HRMS-ESI (+) calcd *m/z* for C_15_H_24_N_3_O^+^ 262.1919 (M+H)^+^, found 262.1925. GCMS 96.36 % purity t_R_=14.15 min.

2.4.30. **U13** (N-((*1R,2R*)-2-(dimethylamino)cyclohexyl)-N-methylbenzamide HCl)

^1^H NMR (400 MHz, METHANOL-*d*_4_) δ ppm 1.34 – 1.56 (m, 2 H), 1.57 – 1.74 (m, 1 H), 1.77 – 1.92 (m, 2 H), 1.97 (br d, *J*=10.96 Hz, 2 H), 2.07 – 2.31 (m, 1 H), 2.91 (s, 3 H), 2.93 (s, 3 H), 2.95 (s, 3 H), 3.71 (td, *J*=11.35, 3.13 Hz, 1 H), 4.76 (m, 1 H), 7.42 – 7.51 (m, 3 H), 7.51 – 7.59 (m, 2 H). HRMS-ESI (+) calcd *m/z* for C_16_H_25_N_2_O^+^ 261.1967 (M+H)^+^, found 261.2018. GCMS >99.00 % purity t_R_=13.75 min.

2.4.31. **U14** (N-((*1R,2R*)-2-(dimethylamino)cyclohexyl)-N-methyl-1-adamantylcarboxamide HCl)

^1^H NMR (400 MHz, METHANOL-*d*_4_) δ ppm 1.29 – 1.47 (m, 2 H), 1.57 (qd, *J*=12.26, 3.52 Hz, 2 H), 1.64 – 1.84 (m, 8 H), 1.90 (br dd, *J*=9.00, 4.30 Hz, 2 H), 1.98 – 2.12 (m, 8 H), 2.13 – 2.29 (m, 1 H), 2.78 (s, 3 H) 2.88 (s, 3 H), 3.16 (s, 3 H), 3.65 (td, *J*=11.44, 3.33 Hz, 1 H), 4.53 – 4.80 (m, 1 H). HRMS-ESI (+) calcd *m/z* for C_20_H_35_N_2_O^+^ 319.2749 (M+H)^+^, found 319.2823. GCMS 98.82 % purity t_R_=16.49 min.

2.4.32. **U15** (N-((*1R,2R*)-2-(dimethylamino)cyclohexyl)-3-chloro-N-methylbenzamide HCl)

^1^H NMR (400 MHz, METHANOL-*d*_4_) δ ppm 1.34 – 1.57 (m, 2 H), 1.57 – 1.73 (m, 1 H), 1.78 – 2.03 (m, 4 H), 2.07 – 2.32 (m, 1 H), 2.90 (s, 3 H), 2.92 (s, 3 H), 2.95 (s, 3 H), 3.70 (td, *J*=11.54, 3.52 Hz, 1 H), 4.72 (m, 1 H), 7.37 – 7.52 (m, 3 H), 7.52 – 7.68 (m, 1 H). HRMS-ESI (+) calcd *m/z* for C_16_H_24_ClN_2_O^+^ 295.1577 (M+H)^+^, found 295.1621. GCMS >99.00 % purity t_R_=14.82 min.

2.4.33. **U16** (N-((*1R,2R*)-2-(dimethylamino)cyclohexyl)-3-fluoro-N-methylbenzamide HCl)

^1^H NMR (400 MHz, METHANOL-*d*_4_) δ ppm 1.34 – 1.57 (m, 2 H), 1.65 (q, *J*=12.26 Hz, 1 H), 1.78 – 2.03 (m, 1 H), 2.23 (br d, *J*=10.56 Hz, 1 H), 2.90 (s, 3 H), 2.92 (s, 3 H), 2.95 (s, 3 H), 3.56 – 3.78 (m, 1 H), 4.74 (m, 1 H), 7.14 – 7.26 (m, 1 H), 7.26 – 7.40 (m, 2 H), 7.41 – 7.57 (m, 1 H). HRMS-ESI (+) calcd *m/z* for C_16_H_24_FN_2_O^+^ 279.1873 (M+H)^+^, found 279.1927. GCMS 97.54 % purity t_R_=13.50 min.

2.4.34. **U17** (N-((*1R,2R*)-2-(dimethylamino)cyclohexyl)-5-chloro-N-methylthienyl-2-carboxamide HCl)

^1^H NMR (400 MHz, METHANOL-*d*_4_) δ ppm 1.34 – 1.54 (m, 2 H), 1.54 – 1.71 (m, 1 H), 1.77 – 2.02 (m, 4 H), 2.10 – 2.35 (m, 1 H), 2.85 (s, 3 H) 2.92 (s, 3 H), 3.23 (s, 3 H), 3.49 – 3.78 (m, 1 H), 4.52 – 4.80 (m, 1 H), 7.02 (d, *J*=3.91 Hz, 1 H), 7.48 (d, *J*=4.30 Hz, 1 H). HRMS-ESI (+) calcd *m/z* for C_14_H_22_ClN_2_OS^+^ 301.1141 (M+H)^+^, found 301.1180. GCMS >99.00 % purity t_R_=14.89 min.

2.4.35. **US01** (N-((*1S,2S*)-2-(dimethylamino)cyclohexyl)-3,4-dichloro-N-methylbenzamide HCl)

^1^H NMR (400 MHz, METHANOL-*d*_4_) δ ppm 1.33 – 1.56 (m, 2 H), 1.56 – 1.72 (m, 1 H), 1.77 – 2.03 (m, 4 H), 2.23 (br d, *J*=10.56 Hz, 1 H), 2.89 (s, 3 H), 2.94 (d, *J*=5.87 Hz, 6 H), 3.70 (td, *J*=11.44, 3.33 Hz, 1 H), 4.72 (m, 1 H), 7.36 – 7.56 (m, 1 H), 7.60 (d, *J*=8.22 Hz, 1 H), 7.70 – 7.88 (m, 1 H). GCMS >99.00 % purity t_R_=16.00 min.

2.4.36. **US02** (N-((*1S,2S*)-2-(dimethylamino)cyclohexyl)-4-chloro-N-methylbenzamide HCl)

^1^H NMR (400 MHz, METHANOL-*d*_4_) δ ppm 1.33 – 1.57 (m, 2 H), 1.57 – 1.72 (m, 1 H), 1.78 – 2.04 (m, 4 H), 2.22 (br d, *J*=12.13 Hz, 1 H), 2.90 (s, 3 H), 2.94 (d, *J*=6.26 Hz, 6 H), 3.58 – 3.81 (m, 1 H), 4.**73** (m, 1 H), 7.46 (d, *J*=8.22 Hz, 2 H), 7.56 (br d, *J*=8.22 Hz, 2 H). GCMS >99.00 % purity t_R_=14.99 min.

2.4.37. **US03** (N-((*1S,2S*)-2-(dimethylamino)cyclohexyl)-4-fluoro-N-methylbenzamide HCl)

^1^H NMR (400 MHz, METHANOL-*d*_4_) δ ppm 1.33 – 1.55 (m, 2 H), 1.55 – 1.72 (m, 1 H), 1.75 – 1.92 (m, 2 H), 1.92 – 2.09 (m, 2 H), 2.23 (br d, *J*=10.96 Hz, 1 H), 2.90 (s, 3 H), 2.94 (s, 6 H), 3.70 (td, *J*=11.54, 3.52 Hz, 1 H), 4.69 – 4.80 (m, 1 H), 7.18 (t, *J*=8.61 Hz, 2 H), 7.42 – 7.69 (m, 2 H). GCMS 98.53 % purity t_R_=13.56 min.

2.4.37. **US04** (N-((*1S,2S*)-2-(dimethylamino)cyclohexyl)-4-trifluoromethyl-N-methylbenzamide HCl)

^1^H NMR (400 MHz, METHANOL-*d*_4_) δ ppm 1.33 – 1.57 (m, 2 H), 1.67 (q, *J*=12.52 Hz, 1 H), 1.78 – 1.94 (m, 2 H), 1.94 – 2.03 (m, 2 H), 2.24 (br d, *J*=10.56 Hz, 1 H), 2.92 (s, 6 H), 2.96 (s, 3 H), 3.72 (td, *J*=11.35, 3.13 Hz, 1 H), 4.56 – 4.80 (m, 1 H), 7.48 – 8.01 (m, 2 H), 7.67 – 7.86 (m, 2 H). GCMS >99.00 % purity t_R_=13.23 min.

2.4.39. **US05** (N-((*1S,2S*)-2-(dimethylamino)cyclohexyl)-4-methoxy-N-methylbenzamide HCl)

^1^H NMR (400 MHz, METHANOL-*d*_4_) δ ppm 1.35 – 1.53 (m, 2 H), 1.62 (br s, 1 H), 1.72 – 1.91 (m, 2 H), 1.91 – 2.09 (m, 2 H), 2.22 (br d, *J*=10.56 Hz, 1 H), 2.89 (s, 3 H), 2.94 (s, 3 H), 2.98 (s, 3 H), 3.66 – 3.78 (m, 1 H), 3.84 (s, 3 H), 4.70 (m, 1 H), 6.98 (d, *J*=8.61 Hz, 2 H), 7.53 (br d, *J*=7.83 Hz, 2 H). GCMS >99.00 % purity t_R_=15.56 min.

2.4.40. **US06** (N-((*1S,2S*)-2-(dimethylamino)cyclohexyl)-3,4-difluoro-N-methylbenzamide HCl)

^1^H NMR (400 MHz, METHANOL-*d*_4_) δ ppm 1.35 – 1.56 (m, 2 H), 1.56 – 1.72 (m, 1 H), 1.77 – 2.04 (m, 4 H), 2.22 (br d, *J*=10.96 Hz, 1 H), 2.89 (s, 3 H), 2.94 (s, 6 H), 3.69 (td, *J*=11.44, 3.72 Hz, 1 H), 4.71 (m, 1 H), 7.27 – 7.48 (m, 2 H), 7.58 (br t, *J*=8.61 Hz, 1 H). GCMS 98.78 % purity t_R_=13.38 min.

2.4.41. **US07** (N-((*1S,2S*)-2-(dimethylamino)cyclohexyl)-3,4-dichloro-N-methylbenzsulfonamide HCl)

^1^H NMR (400 MHz, METHANOL-*d*_4_) δ ppm 0.96 (br d, *J*=10.96 Hz, 1 H), 1.21 – 1.39 (m, 3 H), 1.48 – 1.74 (m, 2 H), 1.86 (br d, *J*=6.26 Hz, 1 H), 2.08 – 2.20 (m, 1 H), 2.82 (s, 3 H), 2.91 (s, 3 H), 2.94 (s, 3 H), 3.36 – 3.54 (m, 1 H), 3.92 – 4.12 (m, 1 H), 7.70 (dd, *J*=8.22, 1.96 Hz, 1 H), 7.75 – 7.85 (m, 1 H), 8.00 – 8.20 (m, 1 H). GCMS >99.00 % purity t_R_=16.60 min.

2.4.42. **US08** (N-((*1S,2S*)-2-(dimethylamino)cyclohexyl)-4-bromo-N-methylbenzamide HCl)

^1^H NMR (400 MHz, METHANOL-*d*_4_) δ ppm 1.29 – 1.54 (m, 2 H), 1.55 – 1.72 (m, 1 H), 1.77 – 2.04 (m, 4 H), 2.22 (br d, *J*=11.35 Hz, 1 H), 2.90 (s, 3 H), 2.92 (s, 3 H), 2.94 (s, 3 H), 3.58 – 3.80 (m, 1 H), 4.53 – 4.80 (m, 1 H), 7.49 (br d, *J*=8.22 Hz, 2 H), 7.63 (d, *J*=8.22 Hz, 2 H). GCMS >99.00 % purity t_R_=15.59 min.

2.4.43. **US09** (N-((*1S,2S*)-2-(dimethylamino)cyclohexyl)-2-chloro-N-methylnicotinamide HCl)

^1^H NMR (400 MHz, METHANOL-*d*_4_) δ ppm 1.30 – 1.58 (m, 2 H), 1.59 – 1.76 (m, 1 H), 1.82 (br s, 1 H), 1.85 – 2.09 (m, 3 H), 2.24 (br d, *J*=12.52 Hz, 1 H), 2.86 (s, 3 H), 2.89 (s, 3 H), 2.94 (s, 3 H), 3.54 – 3.80 (m, 1 H), 4.85 – 5.02 (m, 1 H), 7.49 (dd, *J*=7.63, 4.89 Hz, 1 H), 8.34 (br s, 1 H), 8.44 (dd, *J*=4.89, 1.76 Hz, 1 H). GCMS 95.62 % purity t_R_=15.22 min.

2.4.44. **Udes01** (N-((*1R,2R*)-2-(dimethylamino)cyclohexyl)-3,4-dichlorobenzamide HCl)

^1^H NMR (400 MHz, METHANOL-*d*_4_) δ ppm 1.37 – 1.50 (m, 2 H) 1.53 – 1.70 (m, 2 H) 1.83 (br dd, *J*=6.65, 3.91 Hz, 1 H) 1.92 – 2.08 (m, 2 H) 2.10 – 2.29 (m, 1 H), 2.82 (s, 3 H), 2.91 (s, 3 H), 3.43 (td, *J*=11.54, 3.52 Hz, 1 H) 4.26 (td, *J*=11.15, 4.30 Hz, 1 H) 7.62 (d, *J*=8.61 Hz, 1 H) 7.79 – 7.94 (m, 1 H) 8.12 (d, *J*=1.96 Hz, 1 H). GCMS 97.02 % purity t_R_=15.84 min.

2.4.45. **Udes02** (N-((*1R,2R*)-2-(dimethylamino)cyclohexyl)-4-chlorobenzamide HCl)

^1^H NMR (400 MHz, METHANOL-*d*_4_) δ ppm 1.35 – 1.49 (m, 2 H), 1.51 – 1.70 (m, 2 H), 1.79 – 2.07 (m, 3 H), 2.11 – 2.23 (m, 1 H), 2.82 (s, 3 H), 2.89 (s, 3 H),3.44 (td, *J*=11.54, 3.52 Hz, 1 H), 4.27 (td, *J*=11.35, 4.30 Hz, 1 H), 7.48 (d, *J*=9.00 Hz, 2 H), 7.93 (d, *J*=9.00 Hz, 2 H). GCMS 95.60 % purity t_R_=14.75 min.

2.4.46. **Udes03** (N-((*1R,2R*)-2-(dimethylamino)cyclohexyl)-4-fluorobenzamide HCl)

^1^H NMR (400 MHz, METHANOL-*d*_4_) δ ppm 1.36 – 1.56 (m, 2 H), 1.64 (br d, *J*=11.35 Hz, 1 H), 1.76 – 2.03 (m, 4 H), 2.15 – 2.34 (m, 1 H), 2.89 (s, 2 H), 2.94 (s, 4 H), 3.69 (br t, *J*=10.17 Hz, 1 H), 4.72 (br s, 1 H), 7.23 – 7.47 (m, 2 H), 7.59 (br t, *J*=9.19 Hz, 1 H). GCMS 96.33 % purity t_R_=13.29 min.

2.4.47. **Udes04** (N-((*1R,2R*)-2-(dimethylamino)cyclohexyl)-4-thifluoromethylbenzamide HCl)

^1^H NMR (400 MHz, METHANOL-*d*_4_) δ ppm 1.35 – 1.52 (m, 2 H), 1.54 – 1.76 (m, 2 H), 1.84 (br dd, *J*=6.46, 3.72 Hz, 1 H), 1.91 – 2.09 (m, 2 H), 2.10 – 2.24 (m, 1 H), 2.85 (s, 3 H), 2.91 (s, 3 H), 3.45 (td, *J*=11.54, 3.52 Hz, 1 H), 4.30 (td, *J*=11.15, 4.30 Hz, 1 H), 7.77 (d, *J*=8.22 Hz, 2 H), 8.13 (d, *J*=8.22 Hz, 2 H). GCMS 96.14 % purity t_R_=13.02 min.

2.4.48. **Udes05** (N-((*1R,2R*)-2-(dimethylamino)cyclohexyl)-4-methoxybenzamide HCl)

^1^H NMR (400 MHz, METHANOL-*d*_4_) δ ppm 1.36 – 1.49 (m, 2 H), 1.53 – 1.69 (m, 2 H), 1.76 – 1.90 (m, 1 H), 1.91 – 2.08 (m, 2 H), 2.17 (br d, *J*=14.09 Hz, 1 H), 2.83 (s, 3 H), 2.89 (s, 3 H), 3.32 – 3.56 (m, 1 H), 3.85 (s, 3 H), 4.26 (td, *J*=11.15, 4.30 Hz, 1 H), 6.98 (d, *J*=8.47 Hz, 2 H), 7.91 (d, *J*=9.00 Hz, 2 H). GCMS 97.74 % purity t_R_=15.44 min.

2.4.49. **Udes06** (N-((*1R,2R*)-2-(dimethylamino)cyclohexyl)-3,4-difluorobenzamide HCl)

^1^H NMR (400 MHz, METHANOL-*d*_4_) δ ppm 1.37 – 1.49 (m, 2 H), 1.53 – 1.70 (m, 2 H), 1.75 – 1.88 (m, 1 H), 1.91 – 2.08 (m, 2 H), 2.12 – 2.24 (m, 1 H), 2.83 (s, 3 H), 2.90 (s, 3 H), 3.40 – 3.56 (m, 1 H), 4.26 (td, *J*=11.35, 4.30 Hz, 1 H), 7.27 – 7.44 (m, 1 H), 7.69 – 7.93 (m, 2 H). GCMS 95.67 % purity t_R_=13.13 min.

2.4.50. **Udes07** (N-((*1R,2R*)-2-(dimethylamino)cyclohexyl)-3,4-dichlorobenzsulfonamide HCl)

^1^H NMR (400 MHz, METHANOL-*d*_4_) δ ppm 1.10 – 1.34 (m, 4 H), 1.44 – 1.63 (m, 2 H), 1.71 – 1.88 (m, 1 H), 2.05 – 2.33 (m, 1 H), 2.83 (s, 3 H), 2.90 (s, 3 H), 3.01 – 3.18 (m, 1 H), 3.32 – 3.51 (m, 1 H), 7.64 – 7.75 (m, 1 H), 7.78 – 7.95 (m, 1 H), 8.06 (d, *J*=2.35 Hz, 1 H). GCMS 96.68 % purity t_R_=16.53 min.

2.4.51. **Udes08** (N-((*1R,2R*)-2-(dimethylamino)cyclohexyl)-4-bromobenzamide HCl)

^1^H NMR (400 MHz, METHANOL-*d*_4_) δ ppm 1.29 – 1.57 (m, 4 H), 1.73 – 1.82 (m, 1 H), 1.91 (dt, *J*=6.36, 2.89 Hz, 1 H), 1.99 – 2.13 (m, 2 H), 2.61 (s, 6 H), 2.95 – 3.16 (m, 1 H), 4.02 – 4.25 (m, 1 H), 7.61 (d, *J*=9.00 Hz, 2 H), 7.81 (d, *J*=8.61 Hz, 2 H). GCMS 98.26 % purity t_R_=15.47 min.

2.4.52. **Udes09** (N-((*1R,2R*)-2-(dimethylamino)cyclohexyl)-2-chloronicotinamide HCl)

^1^H NMR (400 MHz, METHANOL-*d*_4_) δ ppm 1.34 – 1.61 (m, 4 H), 1.70 – 1.89 (m, 1 H), 1.89 – 2.05 (m, 1 H), 2.06 – 2.27 (m, 1 H), 2.82 (br s, 6 H), 3.19 (br s, 1 H), 4.06 – 4.26 (m, 1 H), 7.47 (dd, *J*=7.43, 4.70 Hz, 1 H), 8.12 (dd, *J*=7.83, 1.96 Hz, 1 H), 8.45 (dd, *J*=5.09, 1.96 Hz, 1 H). GCMS 96.50 % purity t_R_=14.72 min.
